# Sub-optimal temperature leads to tighter coupling between photosynthetic electron transport and CO_2_ assimilation under fluctuating light in maize

**DOI:** 10.1093/plphys/kiaf581

**Published:** 2025-11-11

**Authors:** Cristina R G Sales, Stéphanie Arrivault, Tomás Tonetti, Vittoria Clapero, Richard L Vath, Lucía Arce Cubas, Mark Stitt, Johannes Kromdijk

**Affiliations:** Department of Plant Sciences, University of Cambridge, Cambridge CB2 3EA, UK; Wild Bioscience, Abingdon OX14 4SA, UK; Max Planck Institute of Molecular Plant Physiology, Am Muehlenberg 1, Potsdam-Golm D-14476, Germany; Instituto de Agrobiotecnología del Litoral (IAL-CONICET), Facultad de Bioquímica y Ciencias Biológicas, Universidad Nacional del Litoral, Santa Fe CP3000, Argentina; Max Planck Institute of Molecular Plant Physiology, Am Muehlenberg 1, Potsdam-Golm D-14476, Germany; Department of Plant Sciences, University of Cambridge, Cambridge CB2 3EA, UK; LI-COR Biosciences, Lincoln, NE 68504, USA; Department of Plant Sciences, University of Cambridge, Cambridge CB2 3EA, UK; Max Planck Institute of Molecular Plant Physiology, Am Muehlenberg 1, Potsdam-Golm D-14476, Germany; Department of Plant Sciences, University of Cambridge, Cambridge CB2 3EA, UK; Institute for Genomic Biology, University of Illinois at Urbana-Champaign, Urbana, IL 61801, USA

## Abstract

The C_4_ carbon concentrating pathway promotes high CO_2_ assimilation rates. To keep C_4_ photosynthesis energetically efficient, electron transport reactions and downstream biochemistry need to be carefully balanced. Here we use a combination of noninvasive measurements and metabolic profiling to study the efficiency of C_4_ photosynthesis in maize (*Zea mays*) under 2 conditions that can lead to decoupling between electron transport and carbon assimilation: fluctuating light and suboptimal temperature. Measurements were performed under 3 fluctuating light regimes and at 3 temperatures, providing the most detailed study to date of the interaction between fluctuating light and suboptimal temperature on the photosynthetic performance of maize, an important global crop. At room temperature, CO_2_ assimilation rates were decoupled from photosynthetic electron transport under fluctuating light regimes, in contrast to tight coordination observed under constant light. This decoupling was underpinned by metabolic flexibility and buffering by large pools of C_4_ transfer metabolites. Surprisingly, at suboptimal temperatures, CO_2_ assimilation rates became more tightly coupled to photosynthetic electron transport rates under fluctuating light regimes. This appeared to be caused by strong feedback downregulation of electron transport and a greater degree of light saturation of CO_2_ assimilation at low temperature. Low temperature impacted carbon assimilation rates more strongly than metabolite pools or intercellular metabolite distribution, which could reflect negative effects on diffusional metabolite transfer through plasmodesmata. Altogether, these results show that maize is able to maintain energetic efficiency by buffering light transitions at room temperature, as well as avoid oxidative damage by strongly downregulating electron transfer under short-term exposure to low temperatures.

## Teaser

Maize CO_2_ assimilation under fluctuating light shows significant decoupling from photosynthetic electron transport at room temperature, but tight coordination is restored under suboptimal temperature.

## Introduction

The C_4_ crop *Zea mays* (maize) was domesticated by ancient farmers in Mexico approximately 9,000 years ago (Matsuoka et al. 2002), and its cultivation has since expanded dramatically, currently being the most widely produced food crop in the world (FAO 2023). C_4_ photosynthesis is an adaptation for hot and dry environments and generally does best under high-light conditions (Sage et al. 1999, 2012). Initial carbon acquisition and subsequent assimilation are spatially separated within the leaves of C_4_ species. In species with “Kranz” anatomy, this involves 2 different photosynthetic cell types, mesophyll cells (MCs), and bundle sheath cells (BSCs), respectively. The initial carbon fixation takes place in MC via carboxylation of phosphoenolpyruvate (PEP). The resulting C_4_ acids diffuse into BSC where they are decarboxylated, elevating the concentration of CO_2_ around ribulose bisphosphate carboxylase oxygenase (Rubisco), which is exclusively expressed in the chloroplasts of BSC. Reduced 3-carbon metabolites diffuse back to MC, and PEP is regenerated at the expense of ATP. The 10- to 100-fold elevation in CO_2_ concentration around Rubisco in BSC of C_4_ plants compared to MC of C_3_ plants (Furbank and Hatch 1987) strongly suppresses ribulose-1,5-bisphosphate (RuBP) oxygenation and associated flux through the photorespiration pathway. However, as a result of the additional energetic expense of the carbon concentration mechanism (CCM), C_4_ plants are typically more strongly light limited across a larger range of light intensities than C_3_ plants (Sales et al. 2021). To maintain light use efficiency, coordination is required between the thylakoid provision of ATP and NADPH and demand by C_4_ cycle and the Calvin–Benson–Bassham (CBB) cycle reactions across both cell types. In nonstressed maize plants, tight coupling is typically evident from significant linear correlations between the quantum yield of Photosystem II (PSII) (Φ_PSII_) and the net rate of CO_2_ assimilation (*A*_CO2_) under a wide range of measurement conditions (e.g. Genty et al. 1989).

Despite the tight coupling observed under nonstressed steady-state conditions, there are 2 environmental scenarios where light-dependent reactions and *A*_CO2_ have been observed to decouple, i.e. deviate from the common linear correlation. Firstly, fluctuating light (FL) can create a temporal mismatch between the thylakoid reactions and downstream biochemistry (reviewed by Kaiser et al. 2015). In C_3_ species, stomatal opening, as well as activity of CBB enzymes such as Rubisco, chloroplastic fructose-1,6-bisphosphatase (FBPase), and sedoheptulose-1,7-bisphosphatase (SBPase), lag behind rapid increases in incident light, leading to impaired assimilation rates compared to those observed under steady-state light. Rapid decreases in light intensity also negatively impact *A*_CO2_ due to the postillumination respiratory burst (Prinsley et al. 1986) and slow relaxation of photoprotective energy quenching (Zhu et al. 2004; Kromdijk et al. 2016). In C_4_ species, it has been proposed that the substantial metabolite pools required for transfer between cell types (Leegood 1985; Stitt and Heldt 1985a,b; Arrivault et al. 2017; Medeiros et al. 2022) may be used as a buffer for ATP and reducing equivalents to supplement the provision from the thylakoid reactions during light fluctuations (Stitt and Zhu 2014), but up to now, this theoretical idea has not been experimentally tested. In addition, the reversible reactions involving 3-phosphoglycerate (3PGA) and triose phosphates (TPs) have been suggested to provide an intercellular buffering system to balance ATP and NADPH demands between MC and BSC (Leegood and von Caemmerer 1988). Both mechanisms may underpin the observed suprasteady-state *A*_CO2_, i.e. net assimilation rates persisting above steady-state rates following high- to low-light transitions observed in C_4_ species across 3 phylogenetically controlled comparisons by Arce Cubas et al. (2023a). However, metabolite sampling during FL treatments would be required to verify if the observed stimulation stems from the hypothesized role of metabolites as a capacitor in C_4_ plants during high-to-low-light transitions. In addition, C_4_ photosynthesis may be less efficient during activation from dark adaptation (Arce Cubas et al. 2023b), or when exposed to rapid increases in light intensity, which may disrupt coordination between the CCM and downstream CBB cycle carbon assimilation leading to incomplete suppression of photorespiration (Kromdijk et al. 2010; Medeiros et al. 2022) or increases in bundle sheath leakiness, i.e. concentrated CO_2_, which subsequently retrodiffuses from BSC to MC (Sage and McKown 2006; Kubásek et al. 2013; Kromdijk et al. 2014; Li et al. 2021; Wang et al. 2022). Both photorespiration and leakiness represent energetic inefficiencies (von Caemmerer and Furbank 1999; Kromdijk et al. 2014) and therefore alter the stoichiometry between photosynthetic electron transfer and CO_2_ assimilation. In a parallel manuscript, we study these phenomena by sampling metabolite timeseries following single increases or decreases in light intensity (Arrivault et al. 2025), whereas the current manuscript focuses on responses to recurring fluctuations.

Secondly, coupling between photosynthetic electron transport and *A*_CO2_ is also altered by exposure to suboptimal temperature. Even though C_4_ photosynthesis should theoretically provide an advantage under cool conditions, and some C_4_ grasses have adapted to cool climates (Du et al. 1999; Long 1999; Long and Spence 2013), C_4_ plants are often particularly sensitive to suboptimal temperature. Temperatures below 15 °C are low enough to cause chilling stress in maize (Hu et al. 2017; Frascaroli and Revilla 2019; Burnett and Kromdijk 2022) and in combination with exposure to light may give rise to chilling-induced photoinhibition (Taylor and Craig 1971; Long 1983). Maize leaves that develop under low temperature tend to show strong decoupling between *A*_CO2_ and linear electron flow. More specifically, strongly increased ratios between electron transport and net CO_2_ assimilation compared to those found in unstressed leaves are typically observed in leaves kept at suboptimal temperature (e.g. Fryer et al. 1998). This may suggest that exposure to suboptimal temperature enhances energy flow to alternative electron sinks to mitigate the decline in carbon assimilation, but the specific mechanisms behind this phenomenon remain unclear. The water–water cycle, whereby linear electron flow from PSII to Photosystem I (PSI), is sustained by reduction of O_2_ and subsequent formation of water, may be elevated in C_4_ grasses (Siebke et al. 2003) and has been suggested to increase under low temperature in both C_3_ and C_4_ species to provide protection against photoinhibition (Ort and Baker 2002). However, experimental verification could not confirm significant flux through the water–water cycle (Driever and Baker 2011). In addition, it is not clear if increases in electron flow per fixed CO_2_ occur in response to short-term low-temperature exposure (hours) and if so, whether these get more pronounced under sharp fluctuations in light intensity, in which case the alternative electron sink may work like a safety valve, by helping to avoid overreduction of electron carriers in the thylakoid membrane.

Tight coordination between the photochemical supply of NADPH and ATP, the C_4_ shuttle, and the CBB cycle carbon assimilation are important features for efficient carbon fixation in C_4_ species (von Caemmerer 2000; Kromdijk et al. 2014), and studying conditions in which this coordination breaks down can help identify prerequisites for efficient C_4_ photosynthesis. Analysis of metabolite profiles provides a means to assess bottlenecks and determine molecular mechanisms underpinning decoupling of CO_2_ assimilation and electron flow. In C_3_ plants, the ratio between 3PGA and dihydroxyacetone phosphate (DHAP) provides a simple proxy for the provision of ATP and reductant to drive phosphorylation and reduction in the CBB cycle (Dietz and Heber 1986). For C_4_ plants, early work by Labate et al. (1990) showed that this ratio decreases with temperature in maize down to 12 °C, but then increased with further decreases in temperature (to 8 °C). This may suggest a restriction in electron transport becomes dominant somewhere between 8 and 12 °C, possibly due to photosynthetic control at the cytochrome *b*_6_*f* complex. However, since 3PGA and DHAP form opposing diffusional gradients between the BSC and MC and 3PGA equilibrates with PEP in the MC, changes in the gradients of 3PGA and DHAP at low temperature may complicate this interpretation in C_4_ plants. Indeed, Labate et al. (1990) observed decreases in whole-leaf 3PGA and DHAP pools, which were hypothesized to reflect a decline in intercellular diffusion between BSC and MC, but experimental verification of MC- and BSC-specific pools at suboptimal temperatures is still lacking.

While current literature shows that both FL and suboptimal temperature can lead to decoupling between photosynthetic electron transport and *A*_CO2_, to our knowledge, there is currently no published data on their combined effects, nor on the metabolic changes that may accompany these conditions and potentially explain nonsteady-state patterns of *A*_CO2_. In this work, we therefore aimed to address these knowledge gaps by studying maize CO_2_ fixation as a function of 3 FL treatments with differing step lengths (6, 30, and 300 s) at ambient and 2 suboptimal temperatures (25, 15, and 7 °C). We specifically hypothesized that:

- decoupling between electron transport and carbon fixation will be more pronounced under light fluctuations with shorter light step lengths;
- after a transition from high to low light, CO_2_ fixation will be transiently supported by energy and reductant from metabolite pools;
- immediately after a transition from low to high light, slow build-up of metabolite pools will cause a lag in CO_2_ fixation;
- short-term exposure to suboptimal temperatures will exacerbate decoupling between electron transport and carbon fixation in line with longer-term acclimatory responses leading to enhancement of alternative electron sinks (e.g. Fryer et al. 1998);
- suboptimal temperature will decrease intercellular metabolite diffusion between BSC and MC as hypothesized by Labate et al. (1990).

To address these hypotheses, we used nonsteady-state leaf gas exchange measurements in conjunction with chlorophyll fluorescence and near-infrared differential absorption to monitor electron transfer efficiencies across the 2 photosystems. In addition, rapid freeze-sampling during gas exchange measurements (Xu et al. 2021) was used to capture metabolite profiles at key timepoints and leaf fractionation by serial filtration over liquid nitrogen was used to resolve distribution of key metabolite pools between M and BSC. Our results reflect substantial metabolic flexibility at 25 °C, but surprisingly showed that coupling between electron transport and *A*_CO2_ was progressively enhanced under low temperature, which we suggest originates both from strong regulation of electron transport to minimize oxidative stress and the strong decrease in CO_2_ assimilation flux under low temperature, which decreases the rebalancing requirements in response to changes in incident irradiance.

## Results

### Low temperature dampens the effect of FL on CO_2_ assimilation

**Figure 1.**
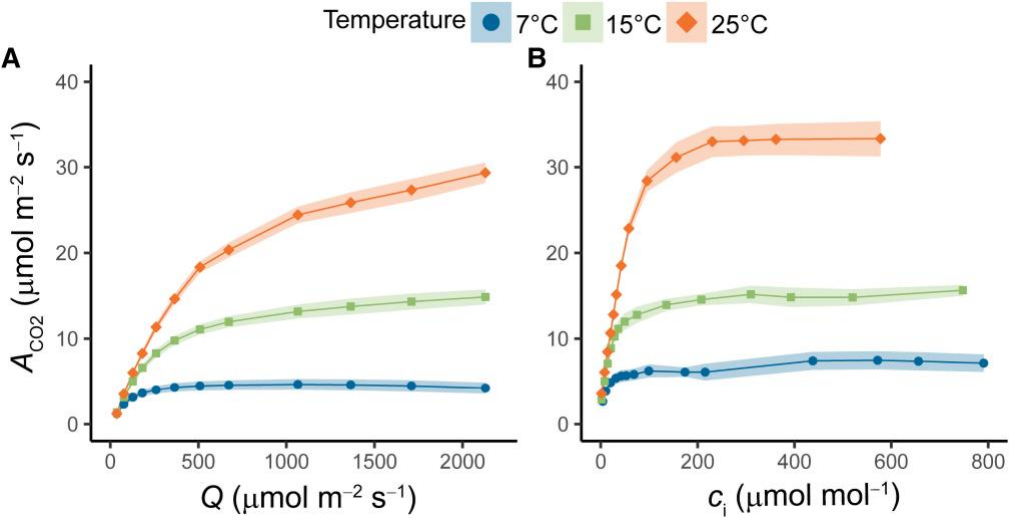
Response curves of net CO_2_ assimilation (*A*_CO2_) in maize leaves measured at different temperatures. **A)** Net CO_2_ assimilation as a function of photosynthetically active radiation (Q) and **B)** net CO_2_ assimilation as a function of *c*_i_. Measurements were performed on maize plants acclimated at 7, 15, or 25 °C for at least 2 h. Ribbons represent standard error of the mean (*n* = 4–5 biological replicates).

Photosynthetic CO_2_ assimilation was first measured under steady-state conditions in maize plants kept at 25, 15, or 7 °C for 2 h (Fig. 1A; Supplementary Table S1). Compared to 25 °C, light-saturated CO_2_ assimilation (*A*_sat_) was reduced by 52% and 86% at 15 °C and 7 °C, respectively. Mitochondrial respiration (*R*_d_) decreased approximately 46% of the rate at 25 °C in plants measured at 15 °C, and the reduction was ∼93% in plants measured at 7 °C. At 15 °C, the maximum quantum yield of CO_2_ assimilation (ϕ_CO2_, (*μ*mol CO_2_ mol^−1^ photons) did not differ significantly from 25 °C (0.062 vs 0.068, respectively) but decreased by 59% when measured at 7 °C (0.039). Finally, the saturation of *A*_CO2_ with light was markedly affected by suboptimal temperature, reaching 95% of the rate at 2,000 *µ*mol m^−2^ s^−1^ PFD at approximately 1,800 *µ*mol m^−2^ s^−1^ for measurements at 25 °C, but at much lower intensities of ∼1,000 and 400 *µ*mol m^−2^ s^−1^ for measurements at 15 and 7 °C respectively. CO_2_ response curves were used to further assess the biochemical limitations of *A*_CO2_ to changes in temperature (Fig. 1B). The maximum in vivo carboxylation rate of phosphoenolpyruvate carboxylase (PEPC) (*V*_pmax_) was reduced by 54% and 84% at 15 °C and 7 °C, respectively, of the rate at 25 °C. Finally, *V*_max_, the CO_2_-saturated rate of photosynthesis, was reduced by 56% at 15 °C and by 80% at 7 °C (Supplementary Table S1).

**Figure 2.**
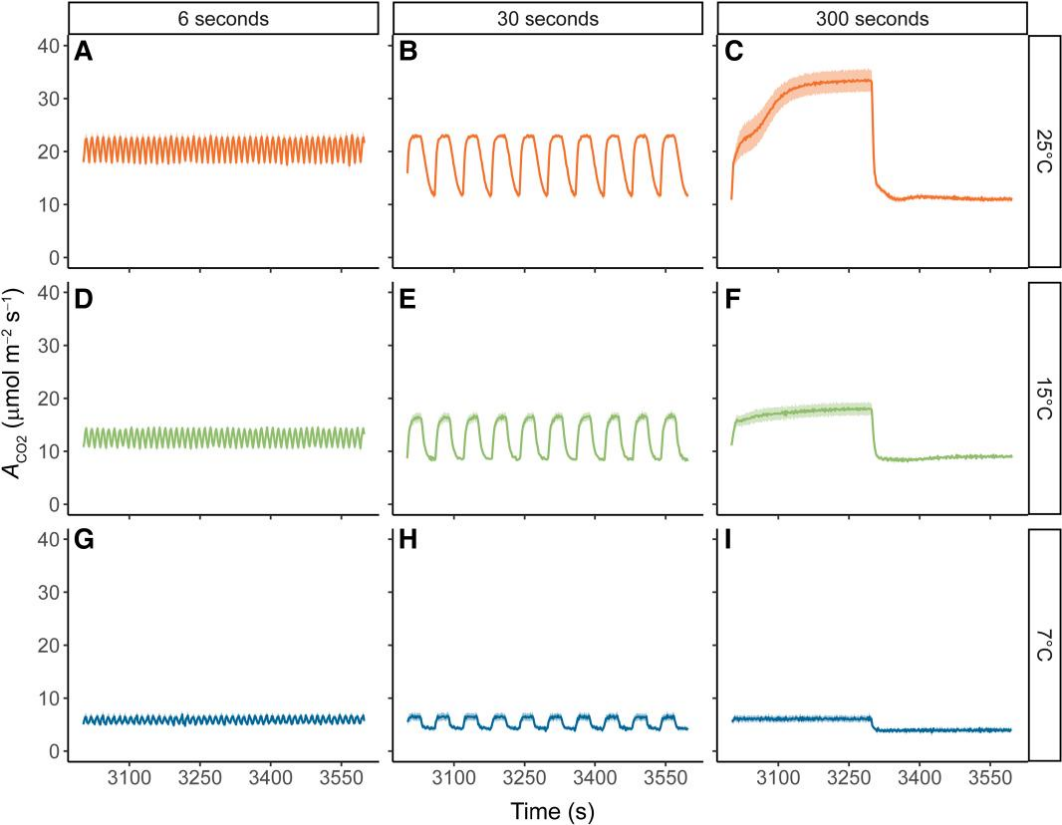
Net CO_2_ assimilation (*A*_CO2_) in maize plants measured under 3 different FL regimes. Measurements were performed at 25 °C **(A** to **C)**, 15 °C **(D** to **F)**, or 7 °C **(G** to **I)**. In each FL regime, leaves were exposed to repetitive changes between low (200 *µ*mol m^−2^ s^−1^) and high (1,500 *µ*mol m^−2^ s^−1^) light steps with a duration of either 6 s (FL6; **A**, **D**, and **G**), 30 s (FL30; **B**, **E**, and **H**) or 300 s (FL300; **C**, **F**, and **I**). Measurements were performed on maize plants acclimated at 7, 15, or 25 °C for at least 2 h. FL regimes were started after leaves were acclimated to steady state at a light intensity of 600 *µ*mol m^−2^ s^−1^ and lasted 1 h. Data are shown from the final ten minutes of each light regime. Ribbons represent standard error of the mean (*n* = 4–5). Full timeseries data are provided in Supplementary Fig. S1, and the timing of the changes in light intensity across each FL regime are provided in Supplementary Fig. S2.

To compare how these temperature effects play out under FL conditions, we measured short-term photosynthetic responses to 3 distinct FL treatments, under the same 3 measurement temperatures. The FL regimes consisted of repetitively switching between high light and low light for 1 h. Photosynthetically active radiation was 1,500 *µ*mol m^−2^ s^−1^ in the high-light phase and 200 *µ*mol m^−2^ s^−1^ in the low-light phase. Each light step lasted either 6, 30, or 300 s, called from now on as FL6, FL30, and FL300 (last 10 min shown in Fig. 2, full hour in Supplementary Fig. S1). As expected, the *A*_CO2_ response showed increases in the 1,500 *µ*mol m^−2^ s^−1^ phase, followed by a decline when light switched to 200 *µ*mol m^−2^ s^−1^. At 25 °C, the difference between *A*_CO2_ values at 1,500 and 200 *µ*mol m^−2^ s^−1^ progressively increased with increasing light step length (Fig. 2, A to C). In addition, following the transition from low to high light in the FL300, *A*_CO2_ showed a biphasic increase with a shoulder around 30 s. A similar shoulder was observed by Arrivault et al. (2025) in response to a single step-change increase in irradiance. When *A*_CO2_ was measured under FL at lower temperatures, the effect of light step length on the difference between *A*_CO2_ values at the 2 light levels decreased for 15 °C (Fig. 2, D to F) and was completely lost at 7 °C (Fig. 2, G to I). The shoulder at 30 s during the FL300 1,500 *µ*mol m^−2^ s^−1^ phase was also lost at lower temperatures (Fig. 2, F and I).

**Figure 3.**
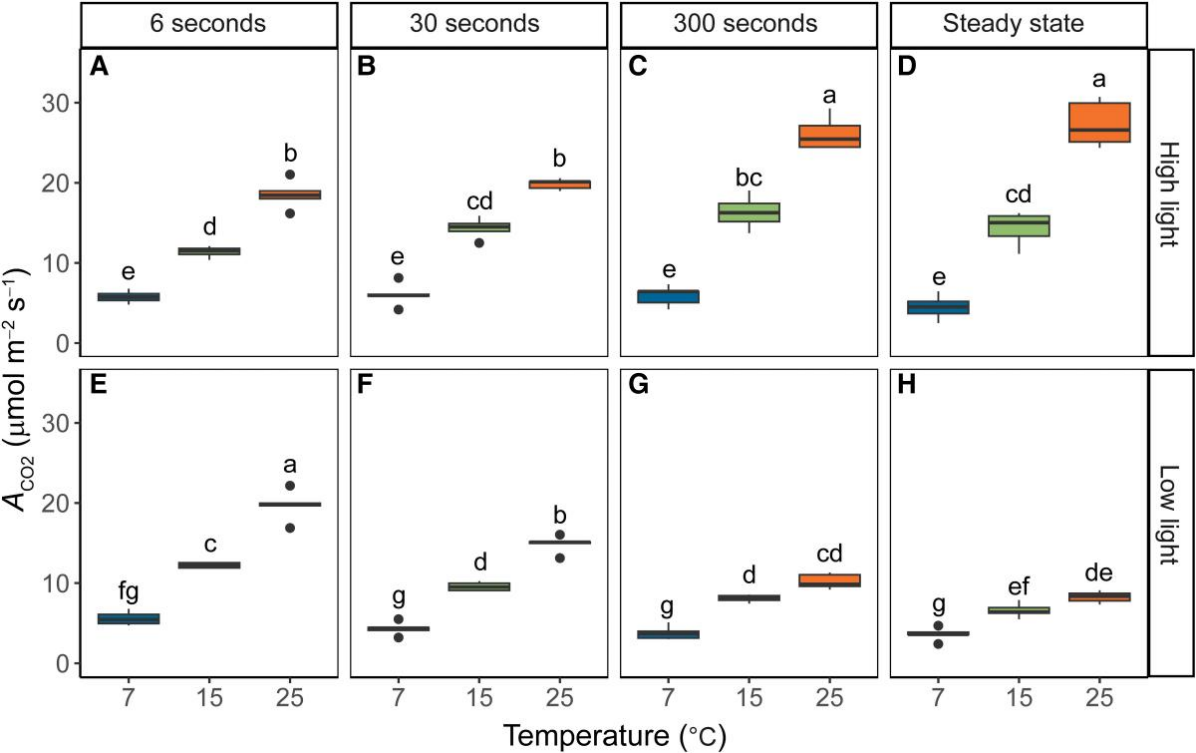
Integrated values of net CO_2_ assimilation (*A*_CO2_) as a function of temperature and FL regime. Measurements were performed at 25, 15, or 7 °C and 3 FL regimes. Measurements were performed on maize plants acclimated at 7, 15, or 25 °C for at least 2 h. In each FL regime, leaves were exposed to repetitive changes between low (200 *µ*mol m^−2^ s^−1^) and high (1,500 *µ*mol m^−2^ s^−1^) light steps with a duration of either 6, 30, or 300 s (FL6, FL30, FL300). Integrated assimilation rates were calculated from the AUC of *A*_CO2_ for each period of high light **(A** to **C)** or low light **(E** to **G)** illumination during the last 10 min of each FL treatment. Numbers were converted to a rate for ease of comparison. Measurements at steady state at matching light intensities and temperatures were obtained from light response curves shown in Fig. 1A and included for comparison **(D** and **H)**. Box edges represent the lower and upper quartiles, the solid line indicates the median, and points represent outliers beyond 1.5 times the interquartile range (*n* = 4–5 biological replicates). Statistical analyses were run on square root transformed data. Two-way ANOVA was used to test the effect of temperature and fluctuation length. Different letters indicate statistical differences according to the Tukey test (*P* < 0.05).

Average values of *A*_CO2_ at high or low light were obtained for the last 10 min cycle for each FL treatment by temperature combination (Fig. 3). To facilitate comparisons, values of *A*_CO2_ under steady state taken from the light response curves (Fig. 1A) at 1,500 and 200 *µ*mol m^−2^ s^−1^ are also shown. At 25 °C, the more rapid FL treatments (FL6 and FL30) caused a significant reduction of ∼30% in *A*_CO2_ at high-light (Fig. 3, A and B) compared to FL300 and steady-state conditions (Fig. 3, C and D). On the other hand, at low-light conditions, the more rapid FL treatments gave rise to the highest integrated *A*_CO2_, with FL6 showing a significant ∼238% increase in *A*_CO2_ (Fig. 3E) compared to steady-state conditions (Fig. 3H). Thus, at 25 °C, higher fluctuation frequency reduced the amplitude of *A*_CO2_ changes between light intensities, with *A*_CO2_ staying intermediate between photosynthetic rates reached in both light conditions at lower fluctuation frequencies. At 15 °C, these patterns were less pronounced, with the most rapid FL treatment (FL6) causing a reduction of ∼20% in *A*_CO2_ at 15 °C (Fig. 3A) compared to FL300 and steady-state conditions (Fig. 3, C and D) at high-light and a ∼185% increase in *A*_CO2_ (Fig. 3E) compared to steady-state conditions (Fig. 3H) at low-light. At 7 °C, the effects of light fluctuations on *A*_CO2_ were fully suppressed, and none of the FL treatments led to significantly different *A*_CO2_ compared to steady-state conditions.

### Low temperature dampens fluctuations in Φ_PSII_ and Φ_PSI_ and enhances coupling between linear electron transport and CO_2_ assimilation

Combined chlorophyll fluorescence and near-infrared differential absorption measurements were used concurrently with the gas exchange measurements in Figs. 1 and 2 to estimate PSII and PSI operating efficiency, both during the steady-state light response curves and at selected timepoints (3 s after the start and 3 s before the end of the high light and 3 s after the start and 3 s before the end of the low light phase; see scheme of measurement timing in Supplementary Fig. S2).

**Figure 4.**
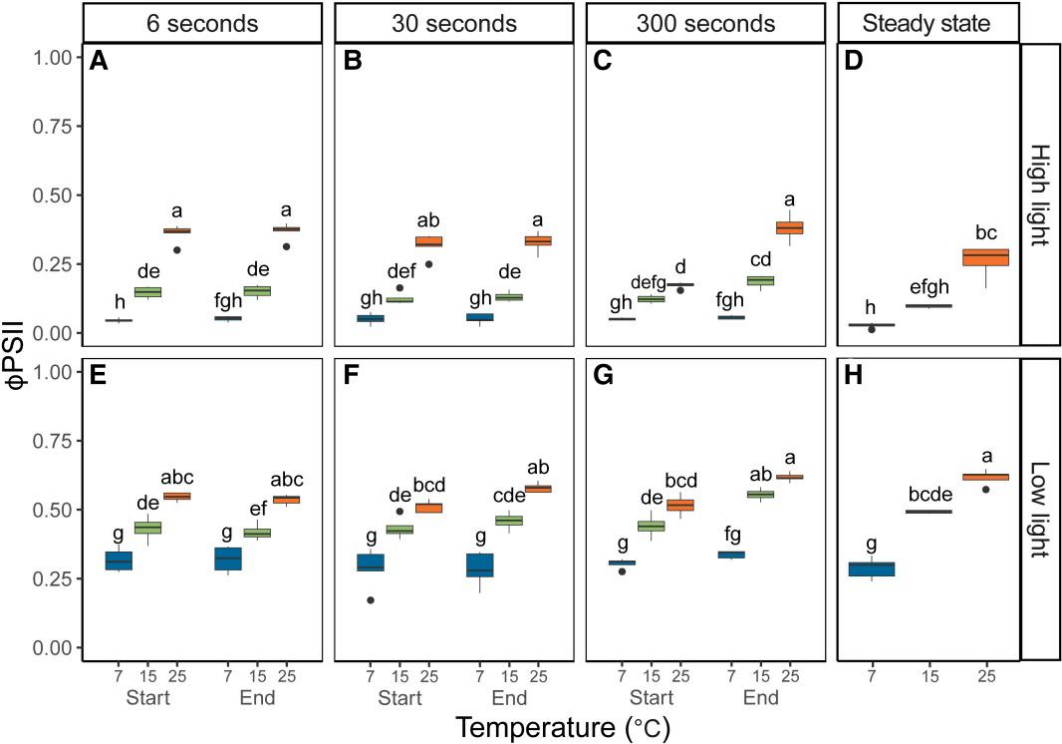
PSII quantum yield (ϕ_PSII_) in maize leaves as a function of temperature and FL regime. Measurements were performed at 25, 15, or 7 °C and 3 FL regimes. Maize plants were acclimated at 7, 15, or 25 °C for at least 2h prior to measurements. In each FL regime, leaves were exposed to repetitive changes between low (200 *µ*mol m^−2^ s^−1^) and high (1,500 *µ*mol m^−2^ s^−1^) light steps with a duration of either 6, 30, or 300 s (FL6, FL30, FL300). In each FL regime, ϕ_PSII_ was determined 3 s into a new light intensity (“Start”) and 3 s before switching (“End”). Thus, a total of 4 measurements were taken for each biological replicate, 2 measurements (“Start” and “End”) during high light **(A** to **C)**, and 2 measurements during low light **(E** to **G)**. Precise timings of measurements in each FL regime are provided in Supplementary Fig. S2. Measurements at steady state at matching light intensities and temperatures were obtained from light response curves shown in Fig. 1A and included for comparison **(D** and **H)**. Box edges represent the lower and upper quartiles, the solid line indicates the median, and points represent outliers beyond 1.5 times the interquartile range (*n* = 4–5 biological replicates). Statistical analyses were run on Box-Cox transformed data. Three-way ANOVA was used to test the effect of temperature, fluctuation length, and measurement time. Different letters indicate statistical differences according to the Tukey test (*P* < 0.05).

Similar to the *A*_CO2_ measurements, Φ_PSII_ responses to the light fluctuations were pronounced at 25 °C, but strongly dampened at 15 °C, and Φ_PSII_ at 7 °C was similar across all measurements performed within each light intensity. Φ_PSII_ measurements were most variable in the FL300 treatment, whereas under more rapid FL regimes (FL6 and FL30), no significant differences were observed in Φ_PSII_ between the start and the end of each light step (Fig. 4, A and B and E and F). At 25 °C, Φ_PSII_ increased significantly from the start to the end of the high light step in the FL300 regime (Fig. 4C). Just after transitioning from low light to high light (labeled “Start”), Φ_PSII_ showed a ∼33% decrease compared to steady-state (Fig. 4C vs 4D). In contrast, the measurement at the end of the high-light step (labeled “End”) showed significantly higher Φ_PSII_ compared to steady state (Fig. 4G vs 4H). Φ_PSII_ can be impacted by the redox state of quinone A (Q_A_), which determines PSII electron acceptor-side limitation and by the level of nonphotochemical quenching (NPQ), which impacts PSII donor-side limitation. At 25 °C, Q_A_ was highly reduced at the “start” timepoint, but less so at the “end” timepoint of the FL300 high light step (Supplementary Fig. S3C), which was also significantly lower than steady state (Supplementary Fig. S3D). In contrast, NPQ was significantly lower at the “start” timepoint than steady state, but increased at the “end” timepoint, which was similar to steady state (Supplementary Fig. S4C vs D). Thus, at 25 °C with increasing duration of high light exposure, a shift from PSII acceptor-side to donor-side limitation was observed. This pattern was similar but dampened under 15 °C, whereas under 7 °C, Q_A_ stayed highly reduced (Supplementary Fig. S3, A to C) and NPQ stayed high and invariable at all timepoints and FL regimes (Supplementary Fig. S4, A to C).

The low-light Φ_PSII_ measurements in the FL300 regime also significantly increased from “start” to “end” at both 15 °C and 25 °C (Fig. 4G), with the “end” measurements indistinguishable from steady state (Fig. 4G vs H). Acceptor-side limitation by reduced Q_A_ was generally low and invariable across the low-light measurements, and therefore the increase in Φ_PSII_ from “start” to “end” predominantly reflected the relaxation of donor-side limitation by NPQ, which started high due to the preceding high-light period and was significantly lower at the “end” than the “start” timepoint (*P* < 0.05; Supplementary Fig. S4, F and G). At 7 °C, low-light Φ_PSII_ measurements were significantly lower than measurements at higher temperatures and were invariable between FL regimes, timepoints, and steady state (Fig. 4, E to H). Consistently, the Q_A_ pool remained approximately 50% reduced throughout all low-light measurements at 7 °C (Supplementary Fig. S3, E to H), and NPQ remained high and became unresponsive to changing light levels in the FL regimes (Supplementary Fig. S4, E to H).

**Figure 5.**
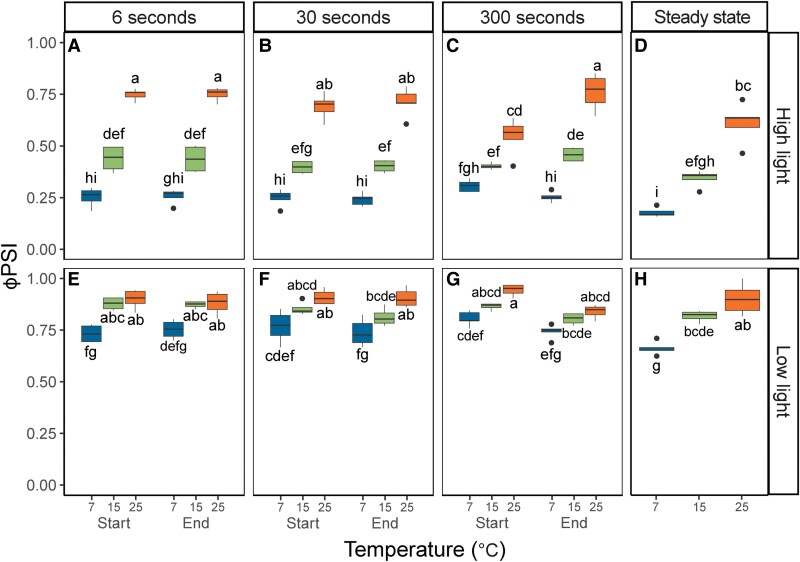
PSI quantum yield (ϕ_PSII_) in maize leaves as a function of temperature and FL regime. Measurements were performed at 25, 15, or 7 °C and 3 FL regimes. Maize plants were acclimated at 7, 15 or 25 °C for at least 2 h prior to measurements. In each FL regime, leaves were exposed to repetitive changes between low (200 *µ*mol m^−2^ s^−1^) and high (1,500 *µ*mol m^−2^ s^−1^) light steps with duration of either 6, 30 or 300 s (FL6, FL30, FL300). In each FL regime, ϕ_PSI_ was determined 3 s into a new light intensity (“Start”) and 3 s before switching (“End”). Thus, a total of 4 measurements were taken for each biological replicate, 2 measurements (“Start” and “End”) during high light **(A** to **C)** and 2 measurements during low light **(E** to **G)**. Precise timings of measurements in each FL regime are provided in Supplementary Fig. S2. Measurements at steady state at matching light intensities and temperatures were obtained from light response curves shown in Fig. 1A and included for comparison **(D** and **H)**. Box edges represent the lower and upper quartiles, the solid line indicates the median, and points represent outliers beyond 1.5 times the interquartile range (*n* = 4–5 biological replicates). Statistical analyses were run on Box-Cox transformed data for high light measurements. Three-way ANOVA was used to test the effect of temperature, fluctuation length, and measurement time. Different letters indicate statistical differences according to the Tukey test (*P* < 0.05).

The operating efficiency of PSI (Φ_PSI_) followed similar patterns as observed for Φ_PSII_ but showed much smaller changes. At 25 °C in the high-light phase, Φ_PSI_ was generally higher than steady state (Fig. 5, A to D), significantly so for both measurements at FL6 treatment (Fig. 5A; *P* < 0.05), as well as the “end” measurement at FL300 (Fig. 5C; *P* < 0.05). These differences did not persist at lower temperatures, where Φ_PSI_ was statistically similar across all FL regimes and timepoints. Electron flow at the donor side of PSI showed a restriction at the cytochrome-b_6,f_-complex as evident from near-infrared differential absorption estimates of plastocyanin redox state, which was fully oxidized throughout all high-light measurements, regardless of temperature (Supplementary Fig. S5, A to D). In contrast, the redox state of ferredoxin was only fully reduced at 7 °C, but significantly more oxidized at 15 and 25 °C (Supplementary Fig. S6, A to D), in line with the increasing demand for NADPH with increasing temperature Under low light, Φ_PSI_ was close to 1 at both 25 and 15 °C, regardless of the FL regime. At 7 °C, Φ_PSI_ was significantly lower than the values observed at higher temperatures and was generally similar in all FL regimes and steady state (Fig. 5, E to H). These patterns could reflect both slower electron flow from plastocyanin to PSI, which was fully oxidized at 7 °C but significantly more reduced at higher temperatures (*P* < 0.05; Supplementary Fig. S5, E to H), and slower electron flow from PSI toward ferredoxin, which was most reduced at 7 °C, but significantly more oxidized at higher temperatures (*P* < 0.05; Supplementary Fig. S6, E to H).

**Figure 6.**
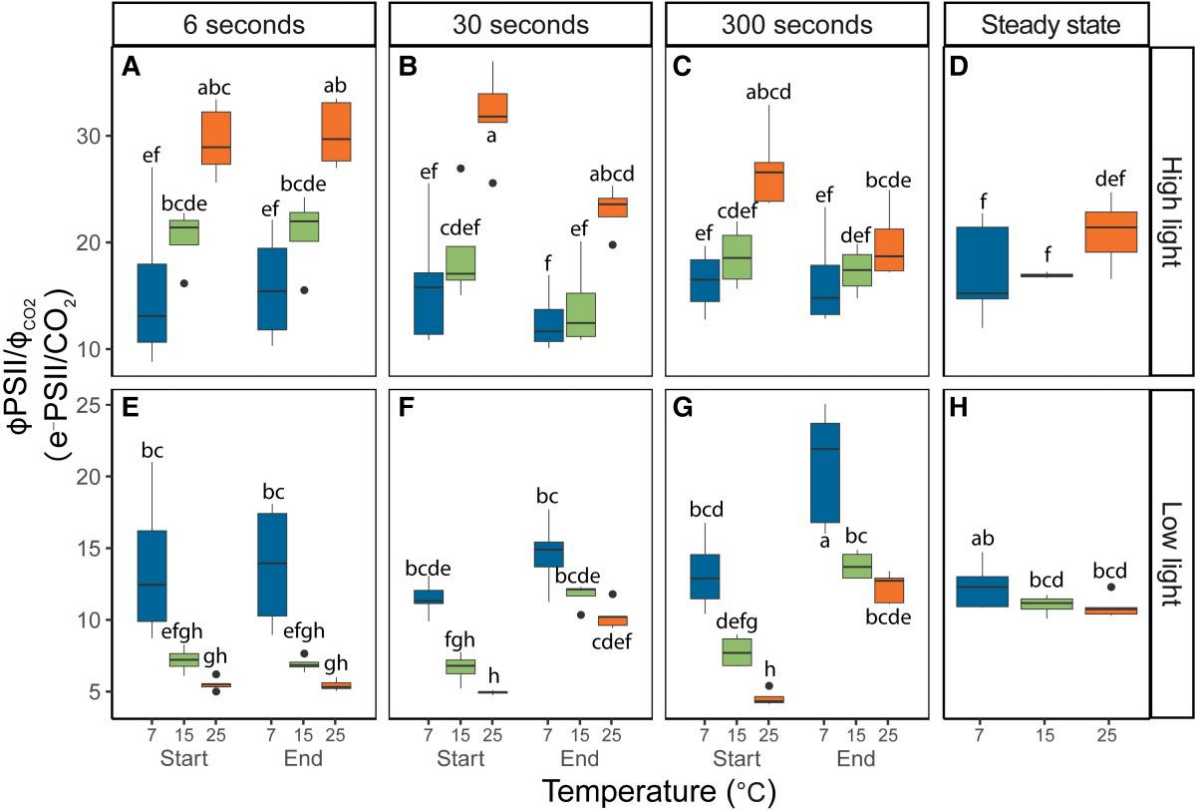
Linear electron transport requirements per fixed CO_2_ (e^−^PSII/CO_2_) in maize leaves as a function of temperature and FL regime. Measurements were performed at 25, 15, or 7 °C and 3 FL regimes. Maize plants were acclimated at 7, 15, or 25 °C for at least 2 h prior to measurements. In each FL regime, leaves were exposed to repetitive changes between low (200 *µ*mol m^−2^ s^−1^) and high (1,500 *µ*mol m^−2^ s^−1^) light steps with a duration of either 6, 30, or 300 s (FL6, FL30, FL300). In each FL regime, ϕ_PSI_ was determined 3 s into a new light intensity (“Start”) and 3 s before switching (“End”). Thus, a total of 4 measurements were taken for each biological replicate, 2 measurements (“Start” and “End”) during high light **(A** to **C)** and 2 measurements during low light **(E** to **G)**. Precise timings of measurements in each FL regime are provided in Supplementary Fig. S2. Measurements at steady state at matching light intensities were obtained from light response curves shown in Fig. 1A and included for comparison **(D** and **H)**. Box edges represent the lower and upper quartiles, the solid line indicates the median, and points represent outliers beyond 1.5 times the interquartile range (*n* = 4–5 biological replicates). Statistical analyses were run on square root transformed data for high light measurements and Box-Cox transformed data for low light measurements. Three-way ANOVA was used to test the effect of temperature, fluctuation length, and measurement time. Different letters indicate statistical differences according to the Tukey test (*P* < 0.05).

The ratio between Φ_PSII_ and Φ_CO2_ calculated from gas exchange at matching timepoints (Supplementary Fig. S7) provides a measure of the number of electrons transferred per CO_2_ fixed (e^−^PSII/CO_2_; Fig. 6), i.e. the degree of coupling between whole-chain electron transport and CO_2_ assimilation (Krall and Edwards 1990). Under steady-state high-light conditions, e^−^PSII/CO_2_ was 17 ± 5 and did not differ significantly between different measurement temperatures (Fig. 6D). In contrast, the impact of temperature was pronounced for the high-light steps in the FL regimes. At 25 °C, e^−^PSII/CO_2_ at the “start” timepoint was significantly higher than steady state (*P* < 0.05) for FL6 (Fig. 6A) and for FL30 (Fig. 6B). Under suboptimal temperatures, high-light e^−^PSII/CO_2_ was significantly higher than steady state (*P* < 0.05) only in the FL6 regime at 15 °C (21 ± 3 e^−^PSII/CO_2_) and was similar to the steady-state ratio across all FL treatments at 7 °C (Fig. 6, A and B). At low light, the steady-state e^−^PSII/CO_2_ ratio was 14 ± 2 electrons per CO_2_ fixed and did not vary with temperature (Fig. 6H). In contrast, at the “start” timepoint of the 25 °C measurements, e^−^PSII/CO_2_ was significantly lower than steady state across all FL regimes at an average value of 5 ± 1 e^−^PSII/CO_2_ (Fig. 6, E to G; *P* < 0.05). At 15 °C, similar but dampened differences were observed, with e^−^PSII/CO_2_ showing average values of 9 ± 3 at the “start” timepoint. At 7 °C, no significant differences were observed between timepoints or FL regimes, with all measurements showing very similar values at 13 ± 3 e^−^PSII/CO_2_ (Fig. 6, E to H). Altogether, these findings show that the tight correlation between electron transport and net CO_2_ fixation is maintained under low-temperature and steady-state light conditions. Nevertheless, in the absence of chilling, the electron requirements per CO_2_ fixed can vary dramatically shortly after a change in light intensity, increasing up to 76% following a change from low to high light in line with the lag in *A*_CO2_ suggested by hypothesis (i) and decreasing by 64% following a change from high to low light in support of metabolic buffering of *A*_CO2_ proposed by hypothesis (ii). Contrary to our hypotheses, these phenomena were only observed at 25 °C, and measurements under suboptimal temperatures showed less variation in e^−^PSII/CO_2_. To find out more about the role of metabolic changes in each of these observations, we next assessed the variation in whole-leaf metabolite profiles.

### Metabolite profiles at 25 °C lag behind changes in light intensity

To further address the hypothesized metabolic origins of the strong decoupling between CO_2_ assimilation and linear electron transport observed primarily at 25 °C (Fig. 6), we determined levels of 25 metabolites involved in the NADP-ME and PEPCK C_4_ pathways, in the CBB cycle, and in the photorespiration pathway, as well as a number of key organic acids, amino acids, and sugars from leaf samples taken at key timepoints during the FL300 regime at 25 °C. Leaves were sampled 10 s prior and following each light switch (i.e. 10 s after light switch from low to high, FL300_10s_HL; 290 s after light switches from low- to high-light phase, FL300_290s_HL; 310 s after light switches from low to light phase, FL300_310s_LL; and 590 s after light switches from low- to high-light phase, FL300_590s_LL). Samples were also taken from steady-state illumination at 200 *µ*mol m^−2^ s^−1^ (low light, LL_Steady-state) or 1,500 *µ*mol m^−2^ s^−1^ (high light, HL_Steady-state).

**Figure 7.**
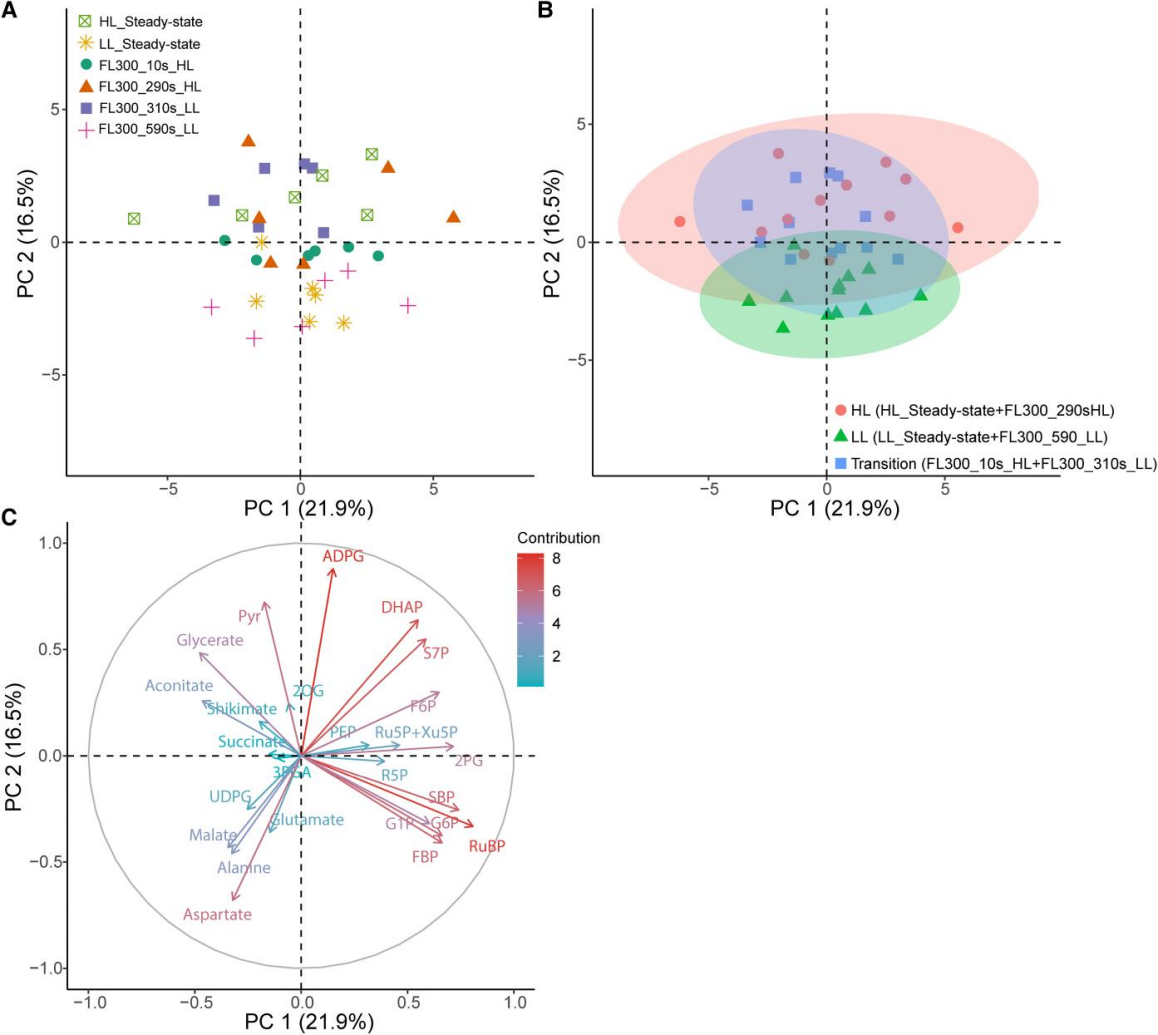
PCA of metabolite profiles of maize leaves exposed to constant or FL. For constant light, leaves were sampled following exposure with low (200 *µ*mol m^−2^ s^−1^) and high (1,500 *µ*mol m^−2^ s^−1^) light intensity. For the FL regime, leaves were exposed to repetitive changes between low and high light steps with a duration of 300 s (FL300). Samples were taken at 4 different timepoints: 10 s after switching from low to high light (FL300_10s_HL), 290 s after switching from low to high light (FL300_290s_HL), 10 s after switching from high to low light (FL300_310s_LL), and 290 s after switching from high to low light (FL300_590s_LL). Plants were acclimated to 25 °C for at least 2 h prior to sampling. Individual sample positions according to the first 2 PCs are shown in **A)**, with different symbols and colors indicating the corresponding light intensity and sampling timepoints. **B)** shows the same sample positions as in **A)** grouped in 3 clusters indicated by different symbols and colors. **C)** shows the different loadings per metabolite. The complete metabolite data are provided in Supplementary Fig. S8.

Nine metabolites varied significantly with sampling timepoint: aspartate, pyruvate (Pyr), PEP, DHAP, sedoheptulose 7-phosphate (S7P), ribulose 5-phosphate + xylulose 5-phosphate (Ru5P + Xu5P), ADP-glucose (ADPG), glycerate, and 2-oxoglutarate (2OG) (*P* < 0.05; Supplementary Fig. S8). Principal component analysis (PCA) showed clustering of the steady-state LL and HL samples at negative and positive values of PC2, respectively (Fig. 7A), with strong positive loadings for Pyr, DHAP, ADPG, glycerate, and S7P and strong negative loadings for aspartate, alanine, malate, FBP, and RuBP (Fig. 7C). Interestingly, positioning along PC2 of the 4 sampled timepoints showed a clear time lag in response to light. Namely, while samples collected just before the end of the 300 s light step (FL300_290s_HL and FL300_590s_LL) clustered with samples taken at steady-state exposure to the same light level (HL and LL, respectively; Fig. 7B), samples taken 10 s after the light switch (FL300_10s_HL and FL300_310s_LL) grouped with the steady-state samples of the opposite light level (LL and HL, respectively), in line with the strong decoupling between linear electron transport and CO_2_ assimilation observed at these timepoints in Fig. 6.

These data were further explored to look for metabolic changes, which may explain substeady-state *A*_CO2_ at FL300_10s_HL (Supplementary Fig. S8Z). Interestingly, the levels of PEP sampled at FL300_10s_HL were lower than steady-state levels, in agreement with what was observed by Arrivault et al. (2025) when maize plants were exposed to a single increase in light intensity. This decrease in PEP might restrict PEPC activity, and in conjunction with low levels of DHAP at FL300_10s_HL, a positive regulator of PEPC activity, suggests that shortfalls both in the substrate supply and in activating metabolites are compounding negatively on PEPC activity.

Metabolite profiles at different timepoints were also scrutinized for changes that may underpin the observed suprasteady-state *A*_CO2_ (Supplementary Fig. S8Z) and decreased e^−^PSII/CO_2_ (Fig. 6, E to H) at FL300_310_LL. Significant increases in 3PGA/PEP were found at timepoint FL300_310s_LL (*P* < 0.05; Supplementary Fig. S9B). Meanwhile, the level of Pyr at FL300_310s_LL remained similar to the preceding FL300_290s_HL high-light timepoint and the steady-state level at high light and was significantly higher than the level at FL300_590s_LL or at steady-state low light (*P* < 0.05; Supplementary Fig. S8C). These patterns of Pyr and 3PGA/PEP indicate that C_4_ cycle activity remained higher than under steady-state low light in the FL300_310s_LL, consistent with significant decoupling between *A*_CO2_ and linear electron transport following the transition from high to low light (Fig. 6). While DHAP/RuBP ratios remained stable (Supplementary Fig. S9C), the 3PGA/DHAP ratio increased from FL300_290s_HL to FL300_590s_LL (Supplementary Fig. S9A), indicating a growing bottleneck of photochemical supply of NADPH, with the FL300_310s_LL timepoint falling between levels at FL300_290s_HL and FL300_590s_LL. Thus, the suprasteady-state levels of CO_2_ assimilation at the FL300_310s_LL may have been supported by provision of 3PGA from DHAP and subsequent interconversion to PEP in MC, resulting in continued substrate provision for C_4_ acid formation. Many of these responses were also observed by Arrivault et al. (2025) when maize plants were exposed to a single increase in light intensity.

The unlabeled metabolite analysis used here, although pragmatic, has some shortcomings. In particular, it does not allow for resolution of the metabolic flux, nor for how much of the observed pools are “inactive,” i.e. do not turn-over as a result of the imposed conditions. Ratios between metabolic pools (Supplementary Fig. S9, A to H) provide a relative measure of changes in the balance between different metabolic pathways, which can be less sensitive to the presence of inactive pools. Since malate is decarboxylated to Pyr in the BSC, we used changes in Asp/Pyr as indirect estimates of relative changes in the aspartate and malate shuttles in response to the light treatments. Under low-light steady-state conditions and at FL300_590s_LL, Asp:Pyr showed a significant increase compared to steady-state high light and to FL300_290s_HL (Supplementary Fig. S9F). When aspartate is moved to the BSC, it must be coupled with return of an amino group to the MC to maintain N stoichiometry. This could happen via transfer of alanine. The Ala:Pyr ratio increased approximately 2-fold with the change from high to low light (Supplementary Fig. S9G), following a similar pattern as Asp:Pyr, in both cases mainly driven by a drop in Pyr (Supplementary Fig. S8C). However, changes in the Asp:Pyr ratio were more pronounced, increasing approximately 4-fold, which may suggest that N stoichiometry was further supported by alternative amino shuttles such as the glutamate:2OG shuttle (Mallmann et al. 2014; Medeiros et al. 2022). Glutamate/2OG (Supplementary Fig. S9H) varied significantly with sampling timepoint and were significantly higher at FL300_590s_LL timepoint compared to the FL300_310s_LL, caused by a significant drop in 2OG concentrations (*P* < 0.05; Supplementary Fig. S8U). Many of these responses were also observed by Arrivault et al. (2025) when maize plants were exposed to a single increase in light intensity.

### Metabolite profiles are strongly affected by low temperature

**Figure 8.**
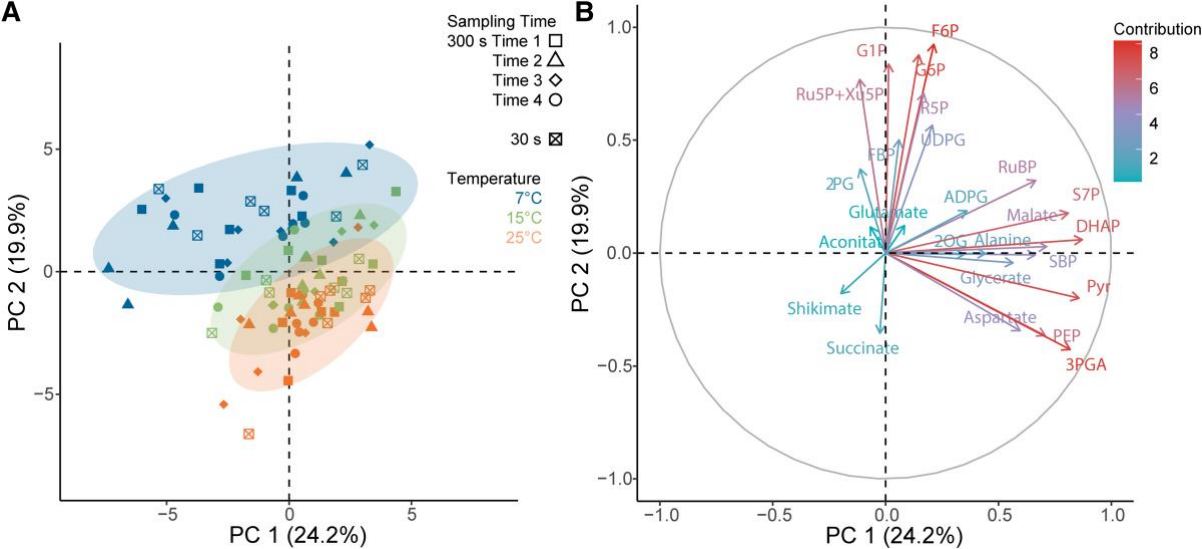
PCA of metabolite profiles of maize leaves exposed to FL regimes at 3 different temperatures. In each FL regime, leaves were exposed to repetitive changes between low (200 *µ*mol m^−2^ s^−1^) and high (1,500 *µ*mol m^−2^ s^−1^) light steps with a duration of either 30 or 300 s (FL30, FL300). For the FL30 light regime, samples were taken 25 s after switching from low to high light (FL30_25s_HL). For the FL300 light regime, samples were taken at 4 different timepoints: 25 s after switching from low to high light (FL300_25s_HL), 290 s after light switches from low to high light phase (FL300_290s_HL), 25 s after switching from high to low light (FL300_325s_LL), and 290 s after switching from high to low light (FL300_590s_LL). Plants were acclimated to 25, 15, or 7 °C for at least 2 h prior to sampling. Individual sample positions according to the first 2 PCs are shown in **A)**, with different symbols indicating different sampling timepoints and different colors representing different temperatures. The loadings of individual metabolites are shown in **B)**. The complete metabolite data are provided in Supplementary Fig. S10.

To find out how different combinations of FL and suboptimal temperature affected metabolite pools, a second experiment was performed where metabolite profiles were sampled across 4 timepoints within the FL300 regime for all 3 temperatures (Supplementary Fig. S10, A to Y). The sampling timepoints were at 25 s after switching from low to high light, to FL300_25s_HL), 290 s after switching from low to high light (FL300_290s_HL), 25 s after switching from high to low light (FL300_325s_LL), and 290 s after switching from high to low light (FL300_590s_LL) and were selected to maximize variation in *A*_CO2_ (Supplementary Fig. S10Z). In addition, we sampled at 25 s after switching from low to high light in the FL30 regime (FL30_25s_HL) to facilitate comparisons with the same sampling timepoint at FL300 (FL300_25s_HL). The effect of temperature on metabolite concentration was significant for 13 metabolites (Temp *P* < 0.05; Supplementary Fig. S10): aspartate, Pyr, PEP, 3PGA, DHAP, fructose 6-phosphate (F6P), S7P, Ru5P + Xu5P, glucose 6-phosphate (G6P), UDP-glucose (UDPG), 2-phosphoglycolate (2PG), glycerate, and succinate, while significant effects of sampling timepoint (Time *P* < 0.05; Supplementary Fig. S10) were found for 8 metabolites: PEP, 3PGA, DHAP, sedoheptulose (SBP), ribose 5-phosphate (R5P), ADPG, 2PG, and 2OG. Significant interactions between temperature and sampling time (Temp × Time *P* < 0.05; Supplementary Fig. S10) were found for only 3 metabolites: PEP, DHAP, and 2PG. PCA (Fig. 8) showed that samples primarily clustered by temperature, aligning diagonally to PCA1 (24%) and PCA2 (20%). The 4 metabolites with the strongest loadings for PC1 were PEP, Pyr, DHAP, and 3PGA (Fig. 8B), which generally showed a decrease under low temperature (Supplementary Fig. S10, C, E, F, and G). The second PC, on the other hand, aligned with variation in many of the phosphorylated sugars, such as F6P, G6P, and glucose 1-phosphate (G1P) (Fig. 8B), which increased under low temperature (Supplementary Fig. S10, H, P, and Q).

Whereas at lower temperatures, CO_2_ fixation was relatively similar to steady state at 25 s following each change in intensity, this was not true for the FL300 regime after the switch to HL at 25 °C where an initial plateau was observed at FL300_25s_HL (Fig. 2C; Supplementary Fig. S10Z). This initial plateau in *A*_CO2_ was similar to peak *A*_CO2_ at FL30_25s_HL (Fig. 2B; Supplementary Fig. S10Z), which is why both timepoints were sampled for metabolites. High levels of PEP were observed at FL30_25s_HL and FL300_25s_HL, compared to significantly lower levels at FL300_290s_HL, FL300_325s_LL, and FL300_590s_LL (Supplementary Fig. S4). These results suggest that a transient bottleneck in the C_4_ cycle due to insufficient carbon availability could have suppressed *A*_CO2_ at FL30_25s_HL and FL300_25s_HL. This interpretation is further supported by the *c*_i_ pattern during these analyses (Supplementary Fig. S11), as both FL30_25s_HL and FL300_25s_HL (Supplementary Fig. S11, B and C) show a drop in intercellular CO_2_ concentration (*c*_i_) values with minima close to 100 *µ*mol mol^−1^. This would transiently be limiting to *A*_CO2_ (see *A*_CO2_/*c*_i_, Fig. 1B) and is subsequently alleviated by stomatal conductance (*g_sw_*; Supplementary Fig. S12), which increases significantly only in FL300 (Supplementary Fig. S12C). Furthermore, consistent with a transient bottleneck in PEP carboxylation, the ratio between 3PGA/PEP (Supplementary Fig. S13B) was also lowest at timepoints FL30_25s_HL and FL300_25s_HL, while the ratios of 3PGA/DHAP and DHAP/RuBP (Supplementary Fig. S13, A and C) were constant across all sampled high-light timepoints showing that flux through the reductive and regenerative parts of the CBB cycle remained tightly coordinated.

### Proportional metabolite distribution between M and BSC is relatively robust under low temperatures

**Figure 9.**
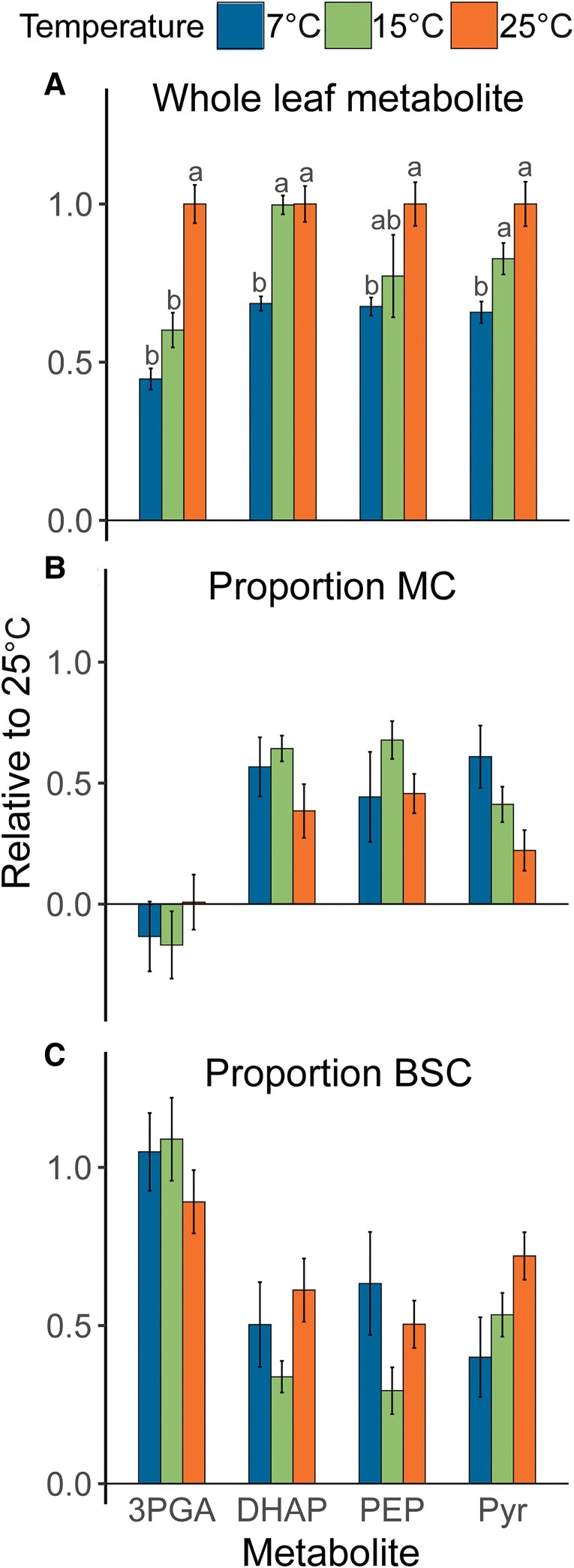
Relative distribution of 3PGA, DHAP, PEP, and Pyr between mesophyll and BSCs in maize leaves exposed to different temperatures. **A)** Decrease of whole-leaf metabolite concentrations at 7 and 15 °C relative to 25 °C. **B)** Relative proportion of whole-leaf metabolite content partitioned in MCs. **C)** Relative proportion of whole-leaf metabolite content partitioned in BSCs. Whole-leaf metabolite contents in **A)** were normalized to 25 °C prior to statistical analysis (in all panels, bars represent means ± SEM; *n* = 5–6 biological replicates). Different letters indicate statistical differences between different temperatures according to the Tukey test (*P* < 0.05). Mesophyll and bundle sheath proportions were estimated from analysis metabolite contents of fractionated leaf samples obtained from serial filtration over liquid nitrogen. Mesophyll and bundle sheath metabolite proportions were estimated from linear regression analysis of metabolite concentrations per fraction as a function of fractional activity of marker enzymes for each cell type. PEPC was used as a marker for MCs and PRK for BSCs. Linear regression analysis is summarized in Supplementary Table S2, marker enzyme activities per leaf fraction are provided in Supplementary Fig. S13, and all regression plots are provided in Supplementary Figs. S14 to S17.

Since C_4_ photosynthesis requires large metabolite pools to drive intercellular metabolite transfer between MC and BSC via diffusion, it is possible that the pronounced low-temperature effects on CO_2_ assimilation are in part driven by collapsed metabolite gradients (Labate et al. 1990). Because whole-leaf variation in PEP, Pyr, DHAP, and 3PGA with temperature strongly determined PC1 in Fig. 8, the effect of low temperature on the proportional distribution of these metabolites between MC and BSC was further characterized. Freeze-sampled leaf material was divided into fractions enriched in MC and BSC via serial filtration of leaf homogenates in liquid N_2_ (Stitt and Heldt 1985a). Metabolite proportions in MC and BSC were estimated by normalizing metabolite concentrations in each fraction against activities of phosphoribulokinase (PRK) or PEPC as markers of BSC and MC, respectively (Supplementary Fig. S13). Whole-leaf metabolite concentrations decreased at 7 °C relative to 25 °C for all 4 metabolites (Fig. 9A). This was most pronounced for 3PGA, which already decreased significantly at 15 °C, whereas DHAP, Pyr, and PEP were not significantly lower at 15 °C, compared to 25 °C. Consistent with previous findings (Stitt and Heldt 1985a,b), 3PGA distribution was highly asymmetric, being entirely partitioned to BSC (Fig. 9C) and undetectable in MC (Fig. 9B) at all 3 temperatures. In contrast, DHAP and PEP proportions were relatively similar between cell types. For Pyr, the distribution was BSC biased at 25 °C, but became more equal at lower temperatures. The higher proportion of total Pyr in BSC at 25 °C is inconsistent with the MC-biased distribution of labeled Pyr observed by Arrivault et al. (2017), but the observations of approximately equal BSC and MC proportions at 15 and 7 °C are similar to earlier findings by Leegood (1985) and Stitt and Heldt (1985a,b) who also found Pyr to be uniformly distributed between both cell types. Whereas the BSC-biased distribution of Pyr at 25 °C shifted to become uniform at 15 and 7 °C, low temperature did not impact the proportional distribution of 3PGA, DHAP, and PEP between M and BSC. As a result, diffusion-driven intercellular metabolite transfer of 3PGA, DHAP, and PEP is unlikely to be impacted by collapsing gradients beyond the effect of a s decrease in gradient size to due to a decline of whole-leaf pool sizes at low temperature (Fig. 9A).

## Discussion

To keep C_4_ photosynthesis energetically efficient, it is important to maintain tight coordination between supply and demand of ATP and reductant. This is evident from highly significant linear correlations between rates of electron transfer and net CO_2_ assimilation, which are typically found in unstressed plants (e.g. Genty et al. 1989). Nevertheless, under FL at 25 °C, this apparent coordination can be lost, which was confirmed here by the strong departures from steady-state e^−^PSII/CO_2_ ranging from −64% under low light to +75% under high light, which were found to be related to distinct metabolic profiles. In addition, we show how these patterns of decoupling are mitigated by suboptimal temperature, with e^−^PSII/CO_2_ becoming essentially invariable with light regimes at the lowest temperature of 7 °C, in contrast to our hypothesis. In the following paragraphs, we discuss how our data provides experimental evidence for metabolic buffering suggested previously, as well as the mechanisms underpinning the effect of low temperatures on maize photosynthesis and CO_2_ fixation as evident from whole-leaf and BSC- and MC-resolved metabolite profiles.

### Metabolic flexibility underpins decoupling between electron transport and CO_2_ fixation at 25 °C

Our experiments demonstrate strong transient decoupling between electron transport and CO_2_ assimilation immediately following a change in light intensity at 25 °C (Fig. 6). At 200 *µ*mol m^−2^ s^−1^ Q, e^−^PSII/CO_2_ decreased from 13 ± 2 at steady-state conditions to 5 ± 1 during the FL6 (Fig. 6E) and FL30 (Fig. 6F) treatments and 3 s after the start of the low-light phase at the FL300 treatment (Fig. 6G, Start). The fact that C_4_ plants are able to sustain CO_2_ fixation above steady-state rates for a short time after a transition from high to low light is consistent with previous studies (Laisk and Edwards 1997; Li et al. 2021; Lee et al. 2022; Arce Cubas et al. 2023a; see also Arrivault et al. 2025). Large pools of C_4_ transfer metabolites and flexibility in decarboxylation routes have been hypothesized to underlie this ability. Energy and redox requirements of CO_2_ fixation could be buffered from large metabolite pools, and flexibility in energy supply and demand between MC and BSC could be met via adjustments to transfer metabolites (Furbank 2011; Stitt and Zhu 2014; Slattery et al. 2018).

Our results provide experimental evidence for some of these hypotheses. Firstly, rapid changes were observed in the relative contribution of aspartate versus malate shuttles (Supplementary Figs. S8B and S9F), with the former getting more pronounced under low light. A shift from malate to aspartate shuttles has been reported previously in maize plants measured following a transition from high to low light (Usuda 1987; Doncaster et al. 1989; Ubierna et al. 2013; Arrivault et al. 2025) as well as in a comparison between steady-state rates at different light intensities (Medeiros et al. 2022) and affects both the redox equivalent moved between MC and BSC and energetic demands of both cell types. Namely, whereas malate transfer moves a redox equivalent from MC to BSC, aspartate transfer does not. And while regeneration of PEP using Pyr from malate decarboxylation requires 2 ATP per PEP, decarboxylation of oxalo-acetate via PEPCK regenerates PEP at only 1 ATP/PEP (Furbank 2011; Bellasio and Griffiths 2014). While the latter may have a quantum yield benefit for the overall pathway (Yin and Struik 2021), another reason suggested to underpin the increased contribution of aspartate at low light is that Pyr movement may require active transport. This was suggested since the intercellular concentration gradient for this metabolite such as observed here for leaves sampled at 25 °C (Fig. 9A) is not consistently found (Stitt and Heldt 1985a; Arrivault et al. 2017). If so, due to the lower supply of ATP during limiting light conditions, Pyr movement could become limiting or less effective in low-light conditions (Medeiros et al. 2022, further discussed in Arrivault et al. 2025).

Secondly, PEP equilibrates with 3PGA via the reversible phosphoglycerate enolase and mutase reactions, which may help to buffer metabolite levels in the CBB cycle and the C_4_ cycle upon changes in light intensity (Huber and Edwards 1975; Furbank and Leegood 1984; Leegood and von Caemmerer 1988; Medeiros et al. 2022). At equilibrium, 3PGA should be approximately 2- to 4-fold higher than PEP levels (Stitt and Heldt 1985a,b; Leegood and von Caemmerer 1988; Ubierna et al. 2013; Medeiros et al. 2022). This agrees well with the observed steady-state ratios around 3.5 (Supplementary Fig. S9B). However, after the transition from high to low light (FL300_310s_LL), the 3PGA/PEP ratio jumped to ∼7.8, which is significantly higher than the steady-state values. Similar changes were also observed by Arrivault et al. (2025) in response to a single step change. Equilibration of isotopic label between 3PGA and PEP was estimated to occur at 18% to 30% of the rate of CO_2_ fixation (Medeiros et al. 2022), which seems consistent with the transient departures from equilibrium ratios observed here. The increase in 3PGA/PEP was mostly due to decreases in PEP (Supplementary Fig. S8E), suggesting that PEP carboxylation continued transiently at suprasteady-state rates following the switch from high to low light, while PEP regeneration from Pyr was downregulated more rapidly. Continued C_4_ cycling may also have driven 3PGA formation in BSC above low-light steady-state rates, in which case spatial separation of MC and BSC pools could further explain the transient increase in 3PGA/PEP ratio. In BSC cells, 3PGA/PEP ratios of up to 20 have been observed (Stitt and Heldt 1985a,b), possibly due to the asymmetric distribution of PGA mutase and enolase activity between cell types, with the major fraction found in MC (Furbank and Leegood 1984).

Thirdly, the reversible CBB cycle reactions between 3PGA and TPs have been suggested to act as a buffer for ATP and NADPH (Stitt and Zhu 2014). Previous measurements of 3PGA/DHAP across increasing irradiance levels showed a steady decrease, inversely proportional to CO_2_ fixation rate (Leegood and Von Caemmerer 1988). Here, a significant increase in the 3PGA/DHAP ratio was observed at the end of the low-light phase (FL300_590s_LL; Supplementary Fig. S9A) compared to the end of the high-light phase (FL300_290s_HL). This increase under limiting light conditions is consistent with other findings (Usuda 1987; Leegood and von Caemmerer 1988; Arrivault et al. 2025) and can be explained by restriction of the conversion of 3PGA to TPs in MC under low light, leading to accumulation of 3PGA. The fact that 3PGA/DHAP gradually increases from FL300_290s_HL to FL300_590s_LL, with FL300_310s_LL showing intermediate values (Supplementary Fig. S9A) while photosynthetic electron transport per unit CO_2_ fixed was significantly below steady state following the switch from high to low light (Fig. 6G), suggests that conversion of DHAP to 3PGA may have contributed to the provision of NADPH and ATP for suprasteady-state CO_2_ fixation at low light during the FL300_310s_LL timepoint (Supplementary Fig. S8Z). Similar changes were also observed by Arrivault et al. (2025) in a more time-resolved analysis of the response to a single step change.

### Low temperature strengthens coupling between electron transport and CO_2_ assimilation during FL

Previous observations of maize plants exposed to chilling conditions showed a strong uncoupling of electron transport and CO_2_ fixation (e.g. Fryer et al. 1998), with rates of electron transport greatly exceeding those predicted by the ATP and NADPH demands of CO_2_ assimilation. On this basis, we hypothesized that short-term exposure to chilling temperature might have a similar effect and potentially aggravate the decoupling caused by FL. Surprisingly, the opposite was found, with chilling temperatures instead negating the decoupling effect of FL (Fig. 6). This suggests that the alternative electron sinks observed previously by Fryer et al. (1998) require longer adaptation to chilling and may require alterations to the pigment pool size and composition in line with recent observations in *Miscanthus* subspecies (Haupt and Głowacka 2024). A requirement for longer chilling exposure would also be consistent with previous work showing that inhibition of CO_2_ assimilation was only marginal after 2 h of low-temperature exposure but became much more pronounced at longer treatment time (Long et al. 1983) in line with the time required for significant de-activation of cold-labile enzymes, such as Pyr orthophosphate dikinase and Rubisco (Sugiyama 1973; Naidu et al. 2003). Thus, despite their inherent cold sensitivity, species such as maize can tolerate short-term exposure to low temperature.

The enhanced coupling observed here under low temperature is remarkable, considering the strong decline in CO_2_ assimilation rate, meaning that the potential mismatch between the absorbed energy to generate NADPH and ATP from the light reactions and the demand for these products in downstream metabolic pathways should have become strongly unbalanced. The fact that this did not lead to uncoupling can be explained by 2 potential mechanisms, which are not mutually exclusive. On the one hand, the negative feedback of lower NADP+ and ADP regeneration via decreases in thylakoid lumen pH would provide a strong trigger to slow down photosynthetic electron transport via induction of sustained NPQ (Supplementary Fig. S4) and downregulation of whole-chain electron transport. The latter is particularly evident in the 7 °C measurements at low light (Fig. 5, E to H), where ϕ_PSI_ does not recover fully, despite the low-light levels. This may reflect a constriction to electron transfer at the Cyt*b*_6_,*f* complex to keep plastocyanin and P700 in oxidized state, as observed previously by Labate et al. (1990), which together with the induction of NPQ would act to prevent runaway ROS formation. Secondly, the chilling temperature had a marked impact on the difference in *A*_CO2_ between high light and low light, which drastically decreased due to saturation of *A*_CO2_ at significantly lower light intensity (Fig. 1A) as evident from the significant lack of reoxidation of ferredoxin (Supplementary Fig. S6, E to H) and Q_A_ (Supplementary Fig. S3, E to H) under low light at 7 °C. As a result, the thylakoid reactions would have to undergo less adjustment following each change in light intensity to accommodate for changes in downstream demand for ATP and NADPH, which instead stayed rather constant between contrasting light levels.

The responses of metabolite levels under FL proposed by hypotheses (ii–iii) were strongly affected by subambient temperature. Metabolite levels varied much less during light fluctuations at 7 °C than at 15 °C or 25 °C, in line with relatively small changes in *A*_CO2_ during light fluctuations at low temperature (Supplementary Fig. S1). In agreement with previous studies (Labate et al. 1990) with a decrease in temperature, 3PGA, PEP, Pyr, and aspartate declined significantly, and a small decrease in DHAP was observed, especially under HL. However, FBP content did not change significantly with temperature. This may reflect coordination of temperature-dependent downregulation of sucrose synthesis. Assuming that FBP follows similar changes to DHAP (as they are linked via aldolase), low temperature may lead to a drop in FBP in the BSC but little change in the MC (Fig. 9). This deduced maintenance of FBP levels in the MC could reflect inhibition of cytosolic FBPase at low temperature. Indeed, cytosolic FBPase is especially inhibited by low temperature in C_3_ plants as it becomes more sensitive to inhibition by fructose 2,6-bisphosphate and adenosine 5′-monophosphate (AMP) as the temperature falls (Stitt and Grosse 1988). It is known that in C_4_ plants, FBPase has a higher Km than in C_3_ species (Stitt and Heldt 1985b). While no studies have been performed for maize FBPase under lower temperature, based on our observations, we suggest that its response is likely similar to those observed in C_3_ isoforms of the enzyme. This would serve to maintain pools of metabolites for the CBB and C4 CCM and contribute to the stability of photosynthesis under FL at low temperature.

### Proportional metabolite distribution between BSC and MC shows only minor impact of temperature

The fact that the C4 CCM requires substantial metabolite transfer between MC and BSC adds complexity to interpretation of responses to FL and chilling temperature. Namely, reaction rates can be affected not only by localized conditions in their respective (sub-) compartment but also by diffusional gradients driving metabolite transfer. At low temperature, photosynthesis rates are often found to be impacted by low availability of inorganic phosphate, due to the accumulation of phosphorylated intermediates caused by slower sucrose synthesis. However, Labate et al. (1990) observed a strong decline in phosphorylated intermediates at low temperature in maize, in contrast with observations in barley, which showed an increase. Our measurements are consistent with the observations by Labate et al. (1990) on maize and suggest that availability of inorganic phosphate did not restrict ATP regeneration. Based on their findings, Labate et al. (1990) hypothesized that the decrease in DHAP reflected a decrease in the rate of 3PGA transfer between MC to BSC and a decline in the diffusional gradient that is required to drive this movement. If so, the equilibration via phosphoglycerate mutase and enolase between 3PGA and PEP might result in decreased PEP in the MC because 3PGA transfer from BSC to MC would decrease under low temperature.

Here, we partially confirm these hypotheses. The diffusional gradient of 3PGA clearly declined under low temperature, but this was entirely due to a decrease in the BSC, since 3PGA in MC remained undetectably low across all conditions (Fig. 9B). Our data did not show evidence of strong equilibration between 3PGA and PEP, since PEP showed a more uniform distribution and whole-leaf pool sizes were less affected by temperature than 3PGA. Similar to PEP and 3PGA, the proportional distribution in DHAP also did not show a clear pattern with temperature, but the diffusional gradients for all 3 metabolites would have decreased with declines in whole-leaf pools, most strongly for 3PGA (Fig. 9A). These findings are consistent with a mechanism whereby at 25 °C 3PGA accumulates in BSC due to a shortfall in NADPH, and the accumulated 3PGA diffuses to the MC where it is reduced. At low temperature, less 3PGA will accumulate due to the negative effect of low temperature on flux in the CBB cycle and Rubisco activity. Lower 3PGA accumulation in BSC in turn should drive a lower rate of diffusion to match the lower rate of photosynthesis. Assuming that transfer of 3PGA and DHAP is fully reliant on diffusion, flux would be expected to be exponentially dependent on absolute temperature and therefore change only moderately if the diffusional gradient is maintained. Of course, this is not the case here, but the diffusional decreases due to absolute temperature (∼6%) and whole-leaf 3PGA concentration (55%) at 7 °C both seem too small to fully explain the strong decrease of 86% in light-saturated CO_2_ assimilation rate (Figs. 1 and 2). We therefore speculate that the latter may reflect additional restrictions under low temperature. It is well-known that plasmodesmata density are strongly enhanced in C_4_ compared to C_3_ leaves (Danila et al. 2019) to facilitate the diffusion of metabolites between mesophyll and BSCs (Hatch and Osmond 1976) and closure of plasmodesmata in maize after 4 h exposure to low temperature by the accumulation of callose and calreticulin has been reported (Bilska and Sowiński 2010). Thus, the disproportionate decrease in CO_2_ assimilation rate under low temperature relative to more moderate changes in metabolite concentration and rate of diffusion may also be partly explained by increased diffusional resistance to metabolite transfer through the plasmodesmata under low temperature. If so, this may also contribute to the strongly dampened amplitude of *A*_CO2_ in response to FL regimes at these temperatures.

## Conclusion

Coordination between NADPH and ATP provision from the thylakoid reactions and flux through the C_4_ and CBB cycles is well-known to be important for efficient C_4_ photosynthesis. Here we investigated the interplay between low temperature and FL, 2 conditions previously suggested to lead to substantial decoupling. Our observations confirm significant decoupling in response to FL, providing evidence for mechanisms of metabolic flexibility, which underpin these departures from steady state, but unexpectedly showed a tighter coupling between electron transfer and CO_2_ fixation at low temperature. We propose that the latter reflects both strong downregulation of electron transport to avoid excessive ROS formation and a stronger degree of light saturation of CO_2_ assimilation at low temperature across both light levels used in our FL regimes, which may have decreased the need for extensive electron transfer adjustment following changes in light. How and when this situation of tight control transitions into the strong decoupling observed in C_4_ leaves following prolonged exposure to suboptimal temperature will be subject for further research.

## Material and methods

### Plant growth conditions

Seeds of maize inbred line B73 (stiff-stalk dent line originating from US Midwest) were sown in Levington Advance M3 compost (Scotts, Ipswich, UK) in seed trays. After 1 wk, seedlings were transplanted to 2 L pots (2 plants per pot), containing a mixture of 2:2:1 of Levington M3 compost:top soil (Westland, Dungannon, UK):perlite 2.0 to 5.0 mm (Sinclair, Ellesmere Port, UK). Each pot was supplemented with 5 g of slow release 17N-9P-11K fertilizer (All Purpose Continuous Release Plant Food, Scotts Miracle-Gro, Marysville, OH, USA), 5 g of magnesium salts (Scotts Miracle-Gro, Marysville, OH, USA), and 10 g of garden lime (Westland, Dungannon, UK). Plants were grown in a Conviron walk-in controlled conditions growth chamber (Conviron Ltd., Winnipeg, MB, CA) at 28/20 °C day/night with a photoperiod of 14 h, photosynthetic photon flux density (*Q*) of 600 *μ*mol m^−2^ s^−1^, and 65% humidity and watered every other day. Plants were grown under these conditions until Stage V5 (fifth completely expanded leaf). Prior to physiological measurements, plants were transferred to a controlled environment chamber (Percival E-41HO, Perry, IA, USA), set up to 25 °C, 15 °C, or 7 °C, and dark acclimated for 1 h before the measurements were performed unless mentioned otherwise.

### Gas exchange measurements

After an hour of dark acclimation at the desired temperature, lights in the cabinet were turned on to a *Q* of 600 *μ*mol m^−2^ s^−1^. Response curves of CO_2_ assimilation rate (*A*_CO2_) to increasing *c*_i_ (*A*_CO2_/*c*_i_ curves), photosynthetic responses to steady light (light response curves; *A*_CO2_/*Q* curves), and to FL conditions were performed on the youngest completely expanded leaf using a Li-6800 portable infrared gas analyzer (IRGA) system (software version 1.4.05, LI-COR, Lincoln, NE, USA) with a 9 cm^2^ leaf chamber (6800-12A) equipped with a 6800-02 light source. For simplicity of aligning illumination treatments across the different instruments, we used 100% red light for actinic illumination. While this may affect stomatal responses to blue light, these are not universal, but instead remarkably species specific (Matthews et al. 2020) and generally much weaker in C4 species such as maize (Zhen and Bugbee 2020; Bernardo et al. 2023). Pilot experiments with 10% blue light also yielded very similar results compared to 100% red light.

For the *A*_CO2_/*c*_i_ and *A*_CO2_/*Q* curves, the conditions inside the chamber were 410 *μ*mol mol^−1^ reference CO_2_ concentration (CO_2__r), 55% relative humidity, and flow rate of 600 *µ*mol s^−1^. Leaf temperature was controlled according to the temperature that plants were measured at: 25 °C, 15 °C, or 7 °C. For the *A*_CO2_/*c*_i_ curves, leaves were acclimated at 1,700 *µ*mol m^−2^ s^−1^ PPFD actinic red light to allow *A*_CO2_ and stomatal conductance (*g*_s_) to reach steady state. Subsequently, gas exchange was measured in the following CO_2__r concentrations: 1,000, 850, 750, 600, 410, 300, 200, 150, 120, 100, 80, 60, 40, 20, and 410 *μ*mol mol^−1^. Gas exchange parameters were logged between 120 and 180 s at each step, and before logging, the reference and sample IRGA signals were matched. The *A*_CO2_/*c*_i_ response curves were fit to a nonrectangular hyperbolic function (von Caemmerer 2000). The initial part of the curve was used to estimate the maximum carboxylation rate of PEPC (*V*_pmax_). A linear model of *A*_CO2_ as a function of *c*_i_ was fitted and the breaking point detected. The response of *A*_CO2_ to *c*_i_ < breaking point was used to solve *V*_pmax_, and *K*_p_, the apparent Michaelis–Menten constant of PEPC for CO_2_, assumed to be 60, 93, and 154 *μ*bar at 7, 15, and 25 °C, respectively (Boyd et al. 2015). The *c*_i_-saturated rate of photosynthesis (*V*_max_) was estimated as the predicted value of each function for *c*_i_ > 2,000 *μ*mol mol^−1^.

For the *A*_CO2_/*Q* curves, leaves were acclimated at 2,100 *µ*mol photons m^−2^ s^−1^ actinic red light to allow *A*_CO2_ and stomatal conductance (*g*_s_) to reach steady state. Incident light intensity was then stepped down through 1,700, 1,350, 1,000, 670, 500, 360, 260, 180, 130, 80, 40, and 0 *µ*mol photons m^−2^ s^−1^. Gas exchange parameters were logged between 60 and 180 s at each step, and before logging, the reference and sample IRGA signals were matched. Light response curves were fitted by a nonrectangular hyperbola (Marshall and Biscoe 1980) to estimate mitochondrial respiration (*R*_d_), maximum *A*_CO2_ assimilation under saturating light (*A*_sat_), convexity (θ), and light compensation point (LCP). Instantaneous quantum efficiency of CO_2_ assimilation (ϕCO_2_) was calculated at each light level as ϕCO_2_ = (*A*_CO2_ + *R*_d_)/α_leaf_, where α_leaf_ is leaf light absorptance measured at the LI6800 light source emission peak (630 nm) with an integrating sphere (Li-1800-12, LI-COR, Lincoln, NE, USA) optically connected to a miniature spectrometer (STS-VIS, Ocean Insight, Orlando, FL, USA) following manufacturer instructions. Leaf absorptance at 630 nm was 0.904 ± 0.002, calculated as α_leaf_ = 1 − *T*_s_ − *R*_s_, where *T*_s_ and *R*_s_ are the leaf transmittance and reflectance, respectively.

To measure photosynthetic responses to FL, leaves were first acclimated at 600 *µ*mol m^−2^ s^−1^ actinic red light, until *A*_CO2_ and *g*_s_ reached constant levels. Using a custom program, leaves were then exposed to repetitive stepwise fluctuations in light intensity from 1,500 to 200 *µ*mol m^−2^ s^−1^  *Q* for 1 h, with gas exchange parameters logged every 2 s. The experiments included 3 different light treatments, with each light step lasting 6, 30, or 300 s. To avoid history effects, only 1 FL treatment by temperature combination was measured per plant, i.e. new plants were used in all cases. To avoid interference with the shorter fluctuations and the data sampling interval, averaging time for head measurements was set to zero, with no additional averaging. Therefore, each log represented an average of the preceding 0.5 s, the inverse of the instrument digital update frequency of 2 Hz. The IRGAs were matched before the FL program started.

As described by Arce Cubas et al. (2023a), a storage flux correction was applied to the measurements under light fluctuations, as they violate the steady-state assumption in default assimilation rate equations. These corrections followed the same principle stated by Saathoff and Welles (2021). Based on the mass balance of the instrument cuvette, the derivative of the cuvette concentration over time can be used to adjust *A*_CO2_ and apparent transpiration rates. A storage flux term for CO_2_ and H_2_O was computed from changes in cuvette concentration between measurements taken every 2 s and applied to adjust *A*_CO2_ and transpiration rates according to Equations 1 and 2, respectively.


(1) $$Storage\;flux_{H_{2}O}=\frac{\frac{PV}{RT}\Delta_{H_{2}O\;}\;}{S\times t}$$

(2) $$Storage\;flux_{CO_{2}}=\frac{-\frac{PV}{RT}\Delta_{CO_{2}\;}\;}{S\times t}$$

In equations 1 and 2, *P* represents pressure (*P*_a_, from instrument), *V* represents cuvette volume (10.72 × 10^−5^ m^3^), *R* represents the molar gas constant, and *T* represents air temperature inside the cuvette to calculate the change in moles of gas (ΔCO_2_ or ΔH_2_O) using instrument recordings of current and preceding logs. *S* represents leaf area (m^2^) and *t* represents the time since last log (s) and was used to convert the molar concentrations to flux per area.

### Dual-KLAS-NIR measurements

A Dual-KLAS-NIR (DKN) analyzer (Heinz Walz GmbH, Effeltrich, Germany) was used in parallel to gas exchange measurements to access chlorophyll fluorescence and P700 redox changes (Klughammer and Schreiber 2016; Schreiber and Klughammer 2016). The DKN was equipped with a gas exchange cuvette (3010-DUAL), connected to a LI-6800 IRGA system (software version 1.4.05, LI-COR, Lincoln, NE, USA) through a custom chamber manifold (6800-19), to control the conditions inside the DKN chamber to a flow rate of 200 *µ*mol s^−1^, CO_2__r of 410 *µ*mol mol^−1^, and 55% relative humidity. Leaf temperature was controlled through a 3010-I/Box with the GFS-Win software (Heinz Walz GmbH, Effeltrich, Germany), according to the temperature that plants were measured: 25 °C, 15 °C, or 7 °C. Before each measurement, plants were dark adapted for 1 h at the desired temperature, and the 4 pairs of pulse-modulated NIR measuring sensors were zeroed and calibrated. Differential model plots (DMPs) were generated, and the maximum oxidation of P700 was determined running the NIRmax script as described by the manufacturer and used to normalize redox-associated absorption changes.

Minimum and maximum dark-adapted fluorescence (*F*_0_ and *F*_m,_ respectively) were determined after 1 h of dark adaptation. During the light response curves and FL responses, chlorophyll fluorescence parameters were determined by using a measuring light intensity of 20 *μ*mol m^−2^ s^−1^ (green measuring light) and a saturating pulse of 25,736 *μ*mol m^−2^ s^−1^. For the PSI redox changes, the measuring light intensity was 14 *μ*mol m^−2^ s^−1^.

The PSII quantum yield (ϕ_PSII_) was calculated as ϕ_PSII_ = (*F*_m_″ − *F*′)/*F*_m_′, where *F*_m_′ is the maximal fluorescence yield from a light-adapted leaf and *F*′ is the steady-state fluorescence yield from leaves under actinic light. The PSI quantum yield (ϕ_PSI_) was calculated from NIR absorptance changes as ϕ_PSI_ = (*P*_m_′ − *P*)/*P*_m_, where *P*_m_ is P700 in the fully oxidized state, *P*_m_′ is the maximum change of the deconvoluted P700 signal from light-adapted leaf after a saturating pulse, and *P* is the steady-state P700 signal. For the steady-state light response curves, flashes were applied after 3 min at each light intensity. For FL measurements, flashes were applied at the beginning of the high-light phase (at 1,803 s for all cycles) and low-light phase (2,103 s for 300 s cycle and 2,133 s for 30 and 6 s cycles); at the end of the high-light phase (2,697 s for 300 s cycle and 2,667 s for 30 and 6 s cycles) and low-light phase (at 2,997 s for all cycles). In all the timepoints described above, flashes were applied 3 s before or after the light switch (see scheme of flashes in Supplementary Fig. S2).

The electron transport rate per assimilated CO_2_ was estimated by the ϕ_PSII_/ϕCO_2_ ratio (Genty et al. 1989; Oberhuber and Edwards 1993), denoted here as e^−^PSII/CO_2_. NPQ was calculated as (*F*_m_  *−*  *F*_m_ʹ)/*F*_m_ʹ. Q_A_ redox state (1 *−*  *q*L) was calculated according to Kramer et al. (2004) where *q*_L_ = (1/*Fʹ*  *−* 1/*F*_m_ʹ)/(1/*F*_o_ʹ *−* 1/*F*_m_ʹ).

### Metabolites

#### Temperature and FL: sampling for metabolite profiles

Plants grown at the same condition and at the same phenological stage as described for physiological measurements were used for metabolite sampling. Plants were acclimated for 1 h at the desired temperature (7, 15, or 25 °C) before starting the light fluctuating cycle. Specific sampling times in both experiments are indicated in Supplementary Figs. S8Z and S10Z.

To ensure accurately timed metabolite sampling, a fast quenching system described by Xu et al. (2021) was used. Briefly, the leaf was enclosed in a LI-6800 IRGA with a 9 cm^2^ leaf chamber (6800-12A) equipped with a 6800-02 light source, set up with the same environmental conditions as described section Gas exchange measurements in Materials and methods. The FL cycle was started, and sampling was performed at least 30 min after the beginning of the program to avoid initial variation in the fluctuation patterns. To quench metabolism at a given timepoint, the 6800-12A leaf temperature thermocouple was removed, and liquid nitrogen was immediately sprayed onto the leaf surface using a cryospray nozzle, through the thermocouple port. The frozen leaf portion was then quickly removed, dropped inside liquid nitrogen, and placed into a precooled microtube. Samples were subsequently stored at −80 °C until metabolite analyses.

#### Temperature: sampling for cell enrichment (gradient)

For the cell separation experiment, plants were acclimated for 1 h at the desired temperature (25, 15, or 7 °C) with the lights in the growth cabinet turned on to a *Q* of 600 *μ*mol m^−2^ s^−1^. Afterward, the youngest completely expanded leaf was clamped into a 9 cm^2^ leaf chamber at 1,500 *µ*mol m^−2^ s^−1^ actinic red light. Gas exchange data was logged every 2 s for 30 min, and metabolism was quenched at steady state after 30 min (Supplementary Fig. S19). Eleven samples from individual plants were pooled together for each cell separation, totaling 6 pooled replicates of 11 plants each. This was needed to provide ∼1 g of fresh weight (FW), which is required for the cell separation protocol. Sampled maize leaves were fractionated as in Stitt and Heldt (1985a,b). Four fractions were obtained by homogenizing ∼1 g FW material and resuspending and filtering sequentially through 200, 80, and 40 *µ*m nylon meshes in the presence of liquid N_2_ (Sefar, Switzerland). Following leaf fractionation, PEP, Pyr, 3PGA, and DHAP concentrations were determined for each fraction (see section c). In addition, the activity of the MC marker enzyme PEPC was measured as in Gibon et al. (2004) with 400-fold dilution (FW/extract volume; Supplementary Fig. S14). For the BSC, the activity of the marker enzyme PRK was measured according to Leegood (1990) with a 200-fold dilution (FW/extract volume; Supplementary Fig. S14). For each metabolite, the ratio of PEPC/PRK (*x* axis) and the ratio metabolite X/PRK (*y* axis) were plotted against each other, and a linear regression was calculated (Supplementary Figs. S15 to S18). The intercept on the *y* axis represented the proportion of metabolite X in the BSCs. If plotted against the alternative marker enzyme, i.e. PRK/PEPC on the *x* axis and the ratio metabolite X/PEPC on the *y* axis, the *y* intercept represented the proportion of metabolite X in the MCs.

#### Metabolite analyses

Maize material was ground to fine powder using a ball mill (Tesch, Haan, Germany) at liquid N_2_ temperature and stored at −80 °C. Samples were analyzed by LC–MS/MS and GC–MS with reference standards for accurate metabolite quantification as in Arrivault et al. (2009). The total amounts of PEP, Pyr, 3PGA, and DHAP were determined enzymatically in freshly prepared trichloroacetic acid extracts as described in Merlo et al. (1993) using a spectrophotometer (Shimadzu, Kyoto, Japan). Alanine was determined enzymatically. A total of 40 *μ*L of extract was added to an assay buffer containing 0.1 m Tris-HCl, pH 10.1, 2 mm EDTA, and 50 mm NAD+. Reactions were performed at 30 °C after adding 0.5 U *μ*L^−1^ of alanine dehydrogenase.

### Statistical analysis

Statistical analyses were performed in R 4.3.2 (R Core Team 2023) on RStudio (2023.12.1, Posit Team 2023). One-way, 2-way, or 3-way ANOVA was used to test the effect of temperature (7 °C vs 15 °C vs 25 °C), fluctuation length (6 s, 30 s, 300 s, and steady state), and measurement time (start or end of the light fluctuation) on the different parameters measured during this study (described in detail on each figure caption). Data for the different traits were tested for homogeneity of variances by Levene's test (α = 0.05) and normality of studentized residual distribution using the Shapiro–Wilk test (α = 0.05). When these tests were not satisfied, variables were transformed prior to ANOVA, or nonparametric tests were applied (as indicated in the figure captions). When ANOVA effects were significant at the 95% confidence level, Tukey post hoc comparisons were used to compare group means. All plots were generated using ggplot2 (Wickham 2016). For light response curve fitting, package *segmented* was used (Muggeo 2008). For the area under the curve (AUC) to obtain integrated *A*_CO2_, bayestestR library was used (Makowski et al. 2019). Package *factoextra* was implemented for PCAs (Kassambara and Mundt 2020).

## Supplementary Material

kiaf581_Supplementary_Data

## Data Availability

All data obtained for this study are presented within the supplementary materials and main manuscript.

## Supplementary data

The following materials are available in the online version of this article.

**Supplementary Table S1.** Parameters estimated from the *A*x*Q* curve and *A*x*c*_i_ curves performed in maize plants acclimated at 7, 15, or 25 °C.

**Supplementary Table S2.** Slopes and intercepts from linear regression analysis of metabolite contents in fractionated leaf tissue.

**Supplementary Figure S1.** Net CO_2_ assimilation (*A*_CO2_) in maize plants measured under 3 different FL regimes.

**Supplementary Figure S2.** Scheme showing timing of chlorophyll fluorescence and P700 redox change measurements in maize plants under 3 different FL regimes.

**Supplementary Figure S3.** Q_A_ redox state in maize leaves as a function of temperature and FL regime.

**Supplementary Figure S4.** NPQ in maize leaves as a function of temperature and FL regime.

**Supplementary Figure S5.** Plastocyanin redox state in maize leaves as a function of temperature and FL regime.

**Supplementary Figure S6.** Ferredoxin redox state in maize leaves as a function of temperature and FL regime.

**Supplementary Figure S7.** Instantaneous quantum yield of CO_2_ fixation (ϕ_CO2_) in maize leaves as a function of temperature and FL regime.

**Supplementary Figure S8.** Metabolite profiles of maize leaves exposed to constant or FL.

**Supplementary Figure S9.** Selected ratios between metabolite profiles of maize leaves exposed to constant or FL.

**Supplementary Figure S10.** Metabolite profiles of maize leaves exposed to FL regimes at 3 different temperatures.

**Supplementary Figure S11.**  *c*_i_ in maize plants measured under 3 different FL regimes.

**Supplementary Figure S12.** Stomatal conductance to water vapor (g_sw_) measured under 3 different FL regimes.

**Supplementary Figure S13.** Selected ratios between metabolite profiles of maize leaves exposed to FL regimes at 3 different temperatures

**Supplementary Figure S14.** Activities of PRK and phosphoenolpyruvate kinase (PEPC) in whole-leaf extract (WL) and in 4 leaf fractions.

**Supplementary Figure S15.** Linear regression analysis of Pyr concentration in 4 leaf fractions.

**Supplementary Figure S16.** Linear regression analysis of phosphoenol-Pyr (PEP) concentration in 4 leaf fractions.

**Supplementary Figure S17.** Linear regression analysis of 3PGA concentration in 4 leaf fractions.

**Supplementary Figure S18.** Linear regression analysis of DHAP concentration in 4 leaf fractions.

**Supplementary Figure S19.** Leaf CO_2_ assimilation (*A*_CO2_) logged before sampling for fractionation of leaf samples.

## Dive Curated Terms

The following phenotypic, genotypic, and functional terms are of significance to the work described in this paper:

PRK Gramene: AT1G32060
PRK Araport: AT1G32060

ATP CHEBI: CHEBI:15422

NADPH CHEBI: CHEBI:16474

metabolite CHEBI: CHEBI:25212

PEPCK Gramene: AT4G37870
PEPCK Araport: AT4G37870
